# Artificial membrane-binding proteins stimulate oxygenation of stem cells during engineering of large cartilage tissue

**DOI:** 10.1038/ncomms8405

**Published:** 2015-06-17

**Authors:** James P. K. Armstrong, Rameen Shakur, Joseph P. Horne, Sally C. Dickinson, Craig T. Armstrong, Katherine Lau, Juned Kadiwala, Robert Lowe, Annela Seddon, Stephen Mann, J. L. Ross Anderson, Adam W. Perriman, Anthony P. Hollander

**Affiliations:** 1 Bristol Centre for Functional Nanomaterials, University of Bristol, Bristol BS8 1FD, UK; 2 Centre for Organized Matter Chemistry and Centre for Protolife Research, School of Chemistry, University of Bristol, Bristol BS8 1TS, UK; 3 School of Cellular and Molecular Medicine, University of Bristol, Bristol BS8 1TD, UK; 4 Wellcome Trust Sanger Institute, Wellcome Trust Genome Campus, Cambridge CB10 1SA, UK; 5 Laboratory for Regenerative Medicine, Department of Surgery, School of Clinical Medicine, University of Cambridge, Cambridge CB2 OQQ, UK; 6 School of Dentistry and Medicine, University of Central Lancashire, Fylde Road, Preston PR1 2HE, UK; 7 School of Biochemistry, University of Bristol, Bristol BS8 1TD, UK; 8 Renishaw plc, Spectroscopy Products Division, Wotton-Under-Edge GL12 7DW, UK; 9 The Blizard Institute, Barts and The London School of Medicine and Dentistry, Queen Mary University of London, London E1 2AT, UK; 10 HH Wills Physics Laboratory, University of Bristol, Bristol BS8 1TL, UK; 11 Present address: Institute of Integrative Biology, Biosciences Building, University of Liverpool, Liverpool L69 7ZB, UK

## Abstract

Restricted oxygen diffusion can result in central cell necrosis in engineered tissue, a problem that is exacerbated when engineering large tissue constructs for clinical application. Here we show that pre-treating human mesenchymal stem cells (hMSCs) with synthetic membrane-active myoglobin-polymer–surfactant complexes can provide a reservoir of oxygen capable of alleviating necrosis at the centre of hyaline cartilage. This is achieved through the development of a new cell functionalization methodology based on polymer–surfactant conjugation, which allows the delivery of functional proteins to the hMSC membrane. This new approach circumvents the need for cell surface engineering using protein chimerization or genetic transfection, and we demonstrate that the surface-modified hMSCs retain their ability to proliferate and to undergo multilineage differentiation. The functionalization technology is facile, versatile and non-disruptive, and in addition to tissue oxygenation, it should have far-reaching application in a host of tissue engineering and cell-based therapies.

## Abstract (web summary)

Avoiding central cell necrosis at the centre of large engineered tissue constructs is an important issue for *in vitro* tissue engineering. Here, the authors demonstrate that this problem may be overcome by oxygenating human mesenchymal stem cells with artificial membrane-binding proteins.

## Graphical abstract

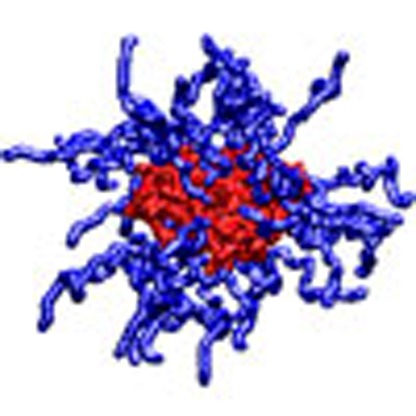

I*n vitro* tissue engineering is emerging as a key strategy for the replacement of damaged or diseased tissue^1^. A typical approach is to immobilize stem cells on a porous scaffold, and induce differentiation and extracellular matrix secretion. However, it has been demonstrated that respiring cells on the scaffold periphery can restrict oxygen availability at the centre of the tissue, leading to areas of cell necrosis and tissue degradation^2^. Moreover, the poor solubility and low diffusion rate of oxygen in aqueous media results in a critical diffusion length of around 200 μm, which severely limits the size of engineered tissue constructs^3^. Mechanical compression^4^ and flow perfusion^5^ can enhance oxygen transport through scaffolds; however, these dynamic culture conditions can negatively impact cell differentiation and tissue formation^6^. Neovascularisation^7^ and tissue printing^8^ strategies allow the engineering of capillary architecture, although these approaches are beneficial only after the engineered tissue integrates with physiological vasculature. A different strategy, which has shown much promise, is the provision of oxygenating species within the tissue construct. For instance, cell viability can be enhanced by using scaffolds impregnated with calcium peroxide, which forms oxygen in a gradual decomposition reaction^9^.

The new approach described herein, however, utilizes the hyperbolic oxygen-binding profile of myoglobin to create a responsive system, with oxygen release triggered only under hypoxic conditions. In this work, a novel polymer–surfactant complex of myoglobin is delivered to the cytoplasmic membrane of stem cells, which are then used to engineer hyaline cartilage constructs with significantly enhanced matrix distribution at the centre of the tissue. This direct cell oxygenation methodology circumvents the size limitations imposed by diffusion limited mass transport and should enable *in vitro* engineering of not only large cartilage constructs, but also other tissues, such as cardiac or bone. Furthermore, this new ‘cell priming' methodology is versatile, and contains the synthetic flexibility to allow it to be readily applied to other proteins and cell types for applications beyond tissue oxygenation, such as cell imaging, homing or signalling.

## Results

### Synthesis of protein–surfactant complexes

Membrane-binding complexes were prepared using enhanced green fluorescent protein (eGFP), to study membrane affinity, and myoglobin (Mb), to promote tissue oxygenation. The reproducible two-step synthesis involved the covalent coupling of acidic amino acid residues with *N,N′*-dimethyl-1,3-propanediamine (DMPA) to form the cationized proteins [eGFP_C] and [Mb_C], which were electrostatically conjugated to the anionic polymer–surfactant glycolic acid ethoxylate 4-nonylphenyl ether (H_19_C_9_-Ph-O-(CH_2_CH_2_O)_40_-CH_2_-COO-, S) to produce the hybrid complexes [eGFP_C][S] and [Mb_C][S]. Cationization efficiencies were evaluated using mass spectrometry (Supplementary Table 1), and corresponded to the addition of 20 and 13 DMPA molecules for [eGFP_C] and [Mb_C], respectively. Zeta potential measurements confirmed that cationization formed a species with positive surface charge, which was neutralized after surfactant conjugation (Supplementary Table 2). Dynamic light scattering measurements revealed a 1–2 nm increase in hydrodynamic radii after surfactant conjugation (Supplementary Table 3), consistent with a protein surrounded by a compact polymer–surfactant corona.

A range of spectroscopic and binding studies were undertaken to elucidate the structure and function of the complexes. Synchrotron radiation circular dichroism (SRCD) spectroscopy experiments revealed that the surface modifications had no effect on the secondary and tertiary structure of [eGFP_C][S], with characteristic β-barrel structural absorbance peaks at 196 and 217 nm (Supplementary Fig. 1a,b). Thermal denaturation melting curves (*λ*=196 nm) performed on [eGFP_C][S] yielded a half denaturation temperature of 73 °C (compared with 76 °C for eGFP), which represents only a slight reduction in thermal stability (Supplementary Fig. 1c). Antibody binding studies provided further evidence of the structural integrity of [eGFP_C][S], with the complexes able to bind anti-GFP functionalized beads (Supplementary Fig. 2). Ultraviolet–visible and fluorescence spectroscopy measurements demonstrated that [eGFP_C][S] retained the native optical function of eGFP, with an absorbance band at 488 nm and a corresponding emission peak at 508 nm (Supplementary Fig. 3). SRCD spectra of [Mb_C][S] showed characteristic α-helical features at 208 and 222 nm, albeit with reduced molar ellipticity when compared with native Mb (Supplementary Fig. 4). Ultraviolet–visible spectroscopy analysis measured ferric, oxyferrous and deoxyferrous Soret bands at 411, 414 and 424 nm, respectively. Interestingly, these peaks more closely resemble neuroglobin (411, 413 and 424 nm)^10^,^11^ than native myoglobin (408, 416 and 433 nm; Supplementary Fig. 5), an observation that can be rationalized by small structural changes in the vicinity of the haem. Significantly, equilibrium oxygen-binding results showed [Mb_C][S] to possess a higher oxygen affinity than native myoglobin, with a dissociation constant of 2.4 × 10^−6^ M (compared with 1.2 × 10^−5^ M for Mb; Supplementary Fig. 6).

### Membrane affinity of protein–surfactant complexes

**Figure 1:**
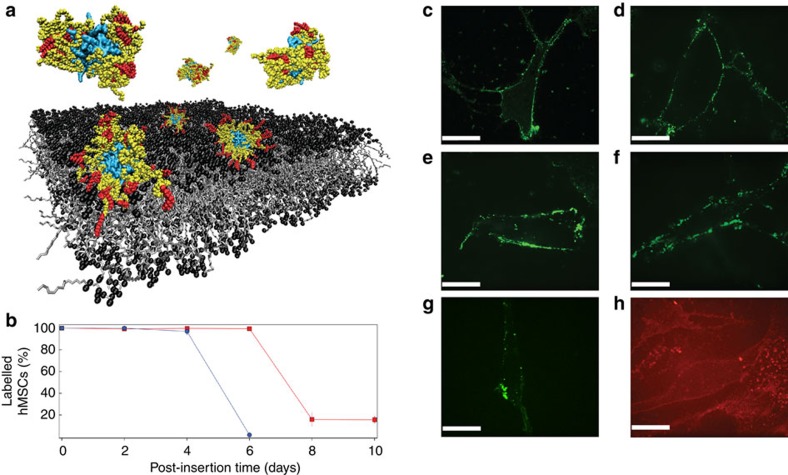
Membrane interactions of the protein–polymer-surfactant complexes. (**a**) Schematic showing that in solution, the polymer–surfactant corona surrounding myoglobin (cyan) adopts a compact conformation with the hydrophobic nonylphenyl tails (red) buried by the poly(ethylene glycol) chains (yellow). Contact with the hydrophobic phospholipid bilayer results in a conformational reorganization of the corona that allows the alkyl chains to anchor the complex to the cell membrane. (**b**) Persistent membrane affinity was demonstrated using flow cytometry. [eGFP_C][S]-labelled hMSCs were observed for up to 10 days in culture (red trace), whereas the membrane-bound [eGFP_C] was cleared within 6 days (blue trace). Live cell confocal fluorescence microscopy images showing [eGFP_C][S] localized at the cytoplasmic membrane of hMSCs (**c**) immediately after priming, (**d**) after 24 h, (**e**) 48 h, (**f**) 72 h and (**g**) 120 h. (**h**) Analogous experiments revealed membrane-bound [Zn-Mb_C][S] immediately after priming. The scale bar in all confocal microscopy images is equal to 25 μm.

The complexes were designed to display an amphiphilic polymer–surfactant corona to facilitate delivery to the cytoplasmic membrane (Fig. 1a). Flow cytometry measurements performed on human mesenchymal stem cells (hMSCs) incubated with 18 μM [eGFP_C][S] at 37 °C showed extensive labelling, while negligible membrane interactions were observed for the native eGFP control (Supplementary Fig. 7). Moreover, further analysis of the depleted supernatant revealed a saturation level of approximately six billion [eGFP_C][S] complexes per cell (Supplementary Fig. 8). Reducing the incubation temperature to 4 °C reduced the labelling efficiency of [eGFP_C][S], while pre-treatment of hMSCs with sodium chlorate, an inhibitor of anionic proteoglycan synthesis, did not affect membrane affinity (Supplementary Fig. 9). These results are consistent with a hydrophobic association mechanism, whereby the nonylphenyl tail within the surfactant corona inserts into the phospholipid membrane bilayer. The intermediate species [eGFP_C] was also capable of labelling hMSCs, albeit in a contrasting mechanism mediated by electrostatic attraction to sulfated proteoglycans, an observation consistent with studies of supercharged proteins^12^. Importantly, the total fluorescence intensity from the [eGFP_C][S]-labelled cells was twofold greater than that from the equivalent [eGFP_C] system (Supplementary Fig. 10), which suggested that there were approximately twice as many functional complexes at the cell membrane. Moreover, hMSCs retained [eGFP_C][S] for up to 10 days in culture; in contrast, [eGFP_C] labelled the hMSCs for <6 days (Fig. 1b). Inhibition assays revealed clathrin-mediated endocytosis to be responsible for the internalization and proteolysis of both [eGFP_C] and [eGFP_C][S] (Supplementary Fig. 11).

Cell viability assays revealed no cytotoxic effects of the priming process (Supplementary Fig. 12) and cell counts showed that labelled hMSCs retained their ability to undergo self-renewal (Supplementary Fig. 13). In addition, the labelled hMSCs were able to undergo adipogenesis, osteogenesis and chondrogenesis (Supplementary Figs 14,15). Live cell confocal fluorescence microscopy was used to observe [eGFP_C][S] at the hMSC cytoplasmic membrane for up to 120 h post incubation (Fig. 1c–g and Supplementary Fig. 16). A three-dimensional image reconstruction revealed discrete clusters of [eGFP_C][S] at the cell membrane, suggesting lateral diffusion and accumulation into clathrin-coated pits, further evidence that the complex is endocytosed by clathrin-mediated endocytosis (Supplementary Fig. 17). Significantly, experiments using a fluorescent myoglobin analogue, [Zn-Mb_C][S], also showed efficient labelling of hMSCs (Fig. 1h). This indicated that the cell functionalization methodology could be readily applied to myoglobin, which was used to alleviate hypoxia during *in vitro* tissue engineering.

### Oxygenation of hMSCs during cartilage tissue engineering

**Figure 2:**
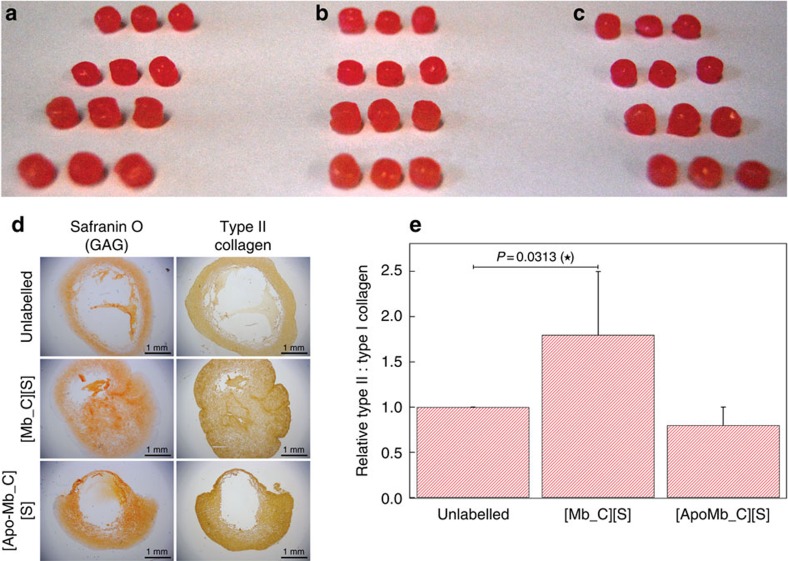
Hyaline cartilage engineering using protein–polymer-surfactant complexes. Constructs of hyaline cartilage were engineered using (**a**) unlabelled hMSCs, (**b**) hMSCs primed with [Mb_C][S] and (**c**) hMSCs primed with [apo-Mb_C][S]. In each case the engineered cartilage was macroscopically similar, with a diameter of ∼5 mm. (**d**) Sections taken from the centre of the cartilage constructs (scale bars equal to 1 mm) were stained for glycosaminoglycan using safranin O, while immunohistochemistry was used to localize type II collagen. Pre-treatment of hMSCs with [Mb_C][S] produced cartilage with significantly decreased central necrosis, compared with the cartilage engineered using unlabelled and [apo-Mb_C][S]-treated hMSCs. (**e**) Enzyme-linked immunosorbent assays on trypsin-digested constructs revealed a significant increase in type II collagen: type I collagen ratio for the cartilage grown using cells primed with [Mb_C][S], while no significant changes were observed in the [apo-Mb_C][S] system. Data is presented as a mean and standard deviation from seven biological replicates. Comparison of differences was measured using Wilcoxon non-parametric paired analysis, with two-tailed *P*-value of <0.05 considered significant and denoted with an asterisk. For full biochemical and histological analysis, please refer to Supplementary Figs 17–19.

Cartilage was selected as a model system, as recent work has demonstrated that severe oxygen gradients present during tissue engineering can impact cell viability and tissue formation^13^. hMSCs were seeded onto large, cylindrical felt discs (5 mm diameter and 6 mm depth) made from polyglycolic acid (PGA), a commonly-used scaffold material for cartilage tissue engineering^14^ (Supplementary Fig. 18). Confocal fluorescence microscopy performed after seeding showed cells distributed throughout the scaffold (Supplementary Fig. 19). The uniformity of seeding was confirmed by RNA measurements, which revealed that the inner 36% of the scaffold contained 32±3% of the hMSCs (Supplementary Fig. 20). Despite this, cartilage engineered using this protocol led to the formation of tissue with poor matrix formation, or even large voids, at the centre of the engineered construct (Fig. 2a–d and Supplementary Fig. 21). Although it is possible that histological processing could have amplified the size of the voids, these observations are consistent with the occurrence of hypoxia-related central cell necrosis. Significantly, pre-treating hMSCs with 18 μM [Mb_C][S] reduced the size of the necrotic core from 42±24 to 7±6% (Supplementary Fig. 22). Furthermore, quantitative biochemical assays revealed that myoglobin priming produced a twofold increase in the type II: type I collagen ratio, a measure of hyaline cartilage quality (Fig. 2e and Supplementary Fig. 23).

**Figure 3:**
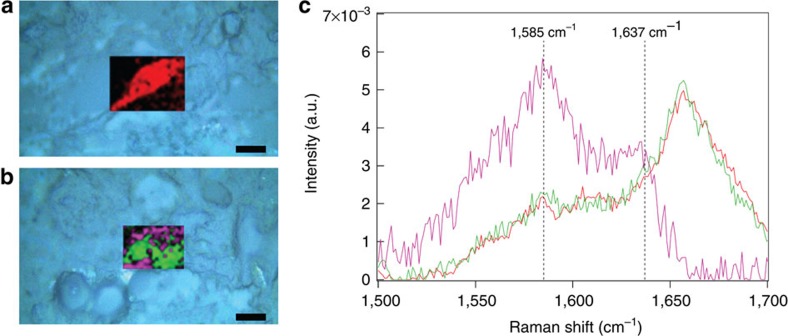
Monitoring the oxidation state of membrane-bound [Mb_C][S]. Raman microscopy scans superimposed over bright field microscopy images (light blue) of ovine mesenchymal stem cells (oMSCs) treated with (**a**) [apo-Mb_C][S] or (**b**) [Mb_C][S] (scale bars equal to 10 μm). oMSCs treated with [apo-Mb_C][S] exhibited a homogenous biochemical profile (red), while the hMSCs treated with [Mb_C][S] revealed two major chemical profiles; one with high intensity at 1,585 cm^−1^ (purple) and another with a profile similar to the [apo-Mb][S] control (green). (**c**) Multivariate curve resolution-alternative least squares (MCR-ALS) analysis was used to deconvolute the Raman spectra from five different cell maps. This revealed that the [Mb_C][S] system possessed a major component with peaks centred at 1,585 and 1,637 cm^−1^ (purple trace). This component corresponded to the spectral fingerprint of oxyferrous myoglobin, which has peaks reported at 1,585 and 1,640 cm^−1^ that are absent in the Raman spectra of deoxyferrous and ferric myoglobin^15^. A background spectral component was recorded for both the [Mb_C][S] and [apo-Mb_C][S] systems (green and red traces, respectively). Taken together, these data are consistent with oxyferrous myoglobin clustered within clathrin-coated pits at the cell membrane.

These observed increases in tissue quality and distribution could be rationalized by the presence of membrane-bound [Mb_C][S] supplying a reservoir of oxygen to cells at the centre of the cartilage construct. This oxygenation hypothesis is supported by the observation that pre-treatment with an analogous [apo-Mb_C][S] complex unable to bind oxygen did not reduce the size of the necrotic core (30±6%), nor enhance the cartilage quality. Raman microscopy measurements on hMSCs labelled with [Mb_C][S] indicated the presence of an oxyferrous haem, with characteristic peaks observed at 1585 and 1637, cm^−1^ (Fig. 3)^15^. With ∼5.9 × 10^9^ oxygenated complexes per cell, each tissue construct would be expected to contain around 7.1 × 10^15^ additional dioxygen molecules. On the basis of consumption rate of 0.8 × 10^−19^ mol cell^−1^ s^−1^ for chondrocytes seeded on polymeric scaffolds^16^, the exogenous myoglobin provides an additional 34 h of oxygen per cell to supplement the environmental oxygen already available to the tissue (Supplementary Methods). Furthermore, the equilibrium binding profile of [Mb_C][S] suggests a net release of oxygen only under extreme hypoxia ([O_2_]<1.4%), corresponding to the oxygen tensions observed within the inner core of cartilage constructs. The expression of known hypoxia genes was measured by quantitative PCR (qPCR) using RNA extracted from the central portion of cartilage engineered with either untreated hMSCs or myoglobin-primed hMSCs (Supplementary Fig. 24). A general trend was observed, whereby the majority of *HIF1α* and downstream hypoxia genes were downregulated in the myoglobin-primed cells after 1 and 7 days of tissue engineering, with significant downregulation observed for the expression of *PRKAA2*, *MB* and *IGFBP1*. This gene analysis suggests that the beneficial effect of priming occurs during the initial stages of chondrogenesis and cartilage formation, which is consistent with the labelling period of 10 days observed for [eGFP_C][S]-labelled hMSCs. After 14 days of tissue engineering, however, many hypoxia genes were significantly upregulated in the myoglobin-primed cells, including *ATP1B1*, *BHLHE40*, *EGLN1*, *EPAS1* and *SOD3*. These network changes, which may be attributed to a dosage compensation effect^17^, could even be responsible for the higher type II: type I collagen ratio produced in the tissue engineered with [Mb_C][S]-primed hMSCs. Given the complexity of hypoxia homoeostasis and adaptation in cells^18^,^19^, it is perhaps not surprising that definable transcriptional changes as a result of treatment may not perturb an individual network of genes in a non-binary and static fashion^20^. For instance, although some biomimetic studies have demonstrated that hypoxia can enhance chondrogenesis and chondrocyte proliferation, these results support a large body of recent work showing that oxygen is an essential nutrient in cartilage development and tissue formation^21^. Moreover, these results mirror recent work showing that ectopic expression of myoglobin in carcinoma cells can reduce oxygen deprivation in experimental tumour models^22^ and that endogenous myoglobin upregulation can act to protect non-muscle tissues against hypoxic stress^23^. The results presented here, however, represent the first example of myoglobin used to alleviate hypoxia in a tissue engineering context.

In conclusion, we report on a new class of protein-polymer–surfactant complexes that possess non-native membrane affinity, which offer a versatile alternative to performing cell surface chemistry^24^, chimerization biotechnology^25^ or genetic manipulation^26^. The methodological flexibility arises from the synthetic design of the surfactant corona, in this case a hydrophobic nonylphenyl tail was utilized to facilitate effective and persistent membrane affinity, while the significant polyethylene oxide segment increased the local dielectric constant and prevented protein denaturation. We have applied this technology to *in vitro* tissue engineering and shown that pre-treating mesenchymal stem cells with myoglobin complexes can improve the tissue distribution and biochemical composition of hyaline cartilage. The myoglobin complexes exhibit a tight, non-linear binding profile, which allows oxygen to remain bound under mild hypoxia, and then release oxygen only when levels are critically low. Here, this technology was utilized to alleviate hypoxia during *in vitro* tissue engineering, and any subsequent implantation would require integration and the adoption of physiological oxygenation mechanisms such as compression-aided convection or vascularization. The versatility of this new cell functionalization process, however, will enable a wide range of functional proteins to be delivered to a variety of cell types. This will have significant impact not only on regenerative medicine, but on a host of cell-based therapies, with potential applications including tracking the migration and integration of transplanted cells using fluorescent proteins, promoting juxtracrine signalling cascades with interactive ligands and targeting tissues or tumours using antibodies.

## Methods

### Protein modification

All chemicals were purchased from Sigma Aldrich, UK, unless stated otherwise. eGFP was recombinantly expressed using BL21-competent *Escherichia coli* and purified using metal ion affinity chromatography. Equine heart Mb was used as purchased, or modified using a 2-butanone solvent extraction and dialysis to produce apo-Mb, followed by reconstitution with protoporphyrin IX zinc (II) in dimethylsulfoxide to yield Zn-Mb. For full details of protein expression and modifications, see Supplementary Methods. Solutions (5 mg ml^−1^) of eGFP, Mb, apo-Mb or Zn-Mb were filtered with a 0.22 μm syringe filter then added to a 100 mg ml^−1^ solution of DMPA at pH 7. The solutions were stirred for 4 h at pH 6.9, and then solid N-(3-Dimethylaminopropyl)-N′-ethylcarbodiimide hydrochloride (EDC) was added and the solution stirred for a further 24 h at pH 6.6 for eGFP and pH 6.0 for Mb, apo-Mb or Zn-Mb. The cationized protein samples were then filtered with a 0.22 μm syringe filter and dialyzed overnight into 20 mM buffer solutions (phosphate buffer at pH 7.0 for [eGFP_C] and HEPES buffer at pH 8.0 for [Mb_C], [apo-Mb_C] and [Zn-Mb_C]). The ratio of DMPA and EDC to the native protein anionic residues was kept consistent at 150 and 34, respectively, to promote high cationization efficiencies. The cationized proteins were conjugated at a ratio of 1.4 surfactant chains per cationic residue, by stirring the protein for 24 h in with a 10 mg ml^−1^ solution of oxidized IGEPAL CO-890 dissolved in 20 mM of the corresponding buffer. For details of surfactant synthesis and the biophysical characterization of modified proteins, see Supplementary Methods.

### Cell culture

hMSCs were harvested from the proximal femur bone marrow of osteoarthritic patients undergoing total hip replacement surgery, in full accordance with Bristol Southmead Hospital Research Ethics Committee guidelines (reference #078/01) and with informed consent from all patients. Ovine mesenchymal stem cells (oMSCs) were obtained from bone marrow of sheep at the Royal Veterinary College (University of London, UK). All cells were cultured at 37 °C and 5% carbon dioxide using expansion media comprised of low glucose (1000, mg dm^−3^) DMEM containing pyridoxine–HCl and NaHCO_3_ with 100 units ml^−1^ penicillin, 100 μg ml^−1^ streptomycin, 2 mM GlutaMAX supplement (Invitrogen, USA), 10% (v/v) foetal bovine serum and 5 ng ml^−1^ freshly supplemented basic human fibroblast growth factor (Peprotech, USA). Media was replenished every 2–3 days and at ∼80% confluency, the cells were passaged using Dulbecco's PBS and trypsin/EDTA solution, up to a passage number of five.

### Cell priming

Expansion media was aspirated and replaced with 18 μM of [eGFP_C][S], [Mb_C][S], [apo-Mb_C][S] and [Zn-Mb_C][S] in 20 mM of the corresponding buffer. Protein–surfactant complex (7 ml) was used for T175 flasks, 1 ml for T25 flasks (Appleton Woods, UK) and 0.5 ml for 35 mm diameter Petri dishes with glass (MatTek, USA) or calcium fluoride (CrysTran Ltd, UK) substrates. The cells were incubated at 37 °C for 30 min, the solution was aspirated, and then the cells were washed using an equivalent volume of 0.04 mg ml^−1^ heparin ammonium salt (Sigma Aldrich, UK) in PBS to remove any unbound complex. The primed cells were then analysed or returned to culture in expansion media at 37 °C. Control experiments were performed using identical conditions with PBS or native and cationized protein analogues. For details of the analysis of the primed cells, including flow cytometry, confocal microscopy and Raman spectroscopy, see Supplementary Methods.

### Cell viability assay

Cytotoxicity was assessed by priming hMSCs with protein–surfactant complex or PBS, culturing the cells at 37 °C for 24 h and then adding CellTiter 96 solution (Promega, USA) to the expansion media for 2 h. The absorbance at 488 nm was measured, and the cell survival of the primed hMSCs were normalized with respect to the PBS-treated cells. For full details of the cell viability assay see Supplementary Methods.

### Proliferation assay

Proliferation was assessed by priming hMSCs with protein–surfactant complex or PBS, culturing the cells at 37 °C for 24 h and then seeding 100,000 cells into T25 flasks. The cells were harvested and counted after 7 or 14 days in culture, and the population doublings of the primed hMSCs were normalized with respect to the PBS-treated cells. For full details of the proliferation assay see Supplementary Methods.

### Differentiation assays

Osteogenesis and adipogenesis were assessed in monolayer by priming hMSCs with protein–surfactant complex or PBS, culturing the cells at 37 °C for 24 h and then inducing differentiation using minimum essential medium containing NaHCO_3_ with 100 units ml^−1^ penicillin, 100 μg ml^−1^ streptomycin, 2 mM GlutaMAX supplement, 10% (v/v) FBS and 50 μl ml^−1^ of freshly-added osteogenic or adipogenic supplement (R&D Systems, UK). The hMSCs were cultured under these conditions for 3 weeks, with media changes twice a week. The hMSCs were then fixed and stained with either Alazarin Red (osteogenesis) or Oil Red (adipogenesis). Chondrogenesis was assessed by priming hMSCs with protein–surfactant complex or PBS, culturing the cells at 37 °C for 24 h, and then engineering cartilage tissue. For full differentiation studies see Supplementary Methods.

### Cartilage tissue engineering

Cartilage tissue engineering was performed using non-woven Biofelt PGA scaffolds with a density of 45 mg cc^−1^, a fibre thickness of 20 μm (Biomedical Structures, USA), a 5 mm diameter and a thickness of either 2 mm (for [eGFP_C][S] chondrogenesis study) or 6 mm (for [Mb_C][S] oxygenation study). The scaffolds were sterilized by ethylene oxide treatment (Anderson Caledonia, UK) and then by a 5 min immersion in 100% ethanol. The scaffolds were washed three times with PBS, immersed for 30 min in 100 μg ml^−1^ fibronectin/PBS solution, dried overnight and added to individual inner wells of a 24-well plate coated with 1 ml of sterile 10 mg ml^−1^ agarose per well. The scaffolds were immersed for 30 min in 1 ml of expansion media, with all bubbles were removed by gently squeezing the scaffolds. The media was completely removed from the scaffold, which was then stood upright and seeded from the top with a 30 μl suspension of 300,000 hMSCs for the 2 × 5 mm scaffolds, or a 90 μl suspension of 1,200,000 hMSCs for the 6 × 5 mm scaffolds. These cells had either been primed with PBS, [eGFP_C][S], [Mb_C][S] or [apo-Mb_C][S] 24 h before seeding. Empty wells of the 24-well plate were filled with sterile antifungal water containing 100 units ml^−1^ penicillin, 100 μg ml^−1^ streptomycin and 2.5 μg ml^−1^ amphotericin B, and then the plate was incubated overnight at 37 °C. The scaffolds were then turned over and returned to the incubator for 2 h at 37 °C.

Differentiation was induced through the addition of chondrogenic media comprising high glucose (4,500 mg dm^−3^) DMEM containing pyridoxine–HCl and NaHCO_3_ with 100 units ml^−1^ penicillin, 100 μg ml^−1^ streptomycin, 2 mM GlutaMAX supplement, 1% (v/v) insulin transferrin selenium solution, 1 mM sodium pyruvate, freshly supplemented with 100 nM dexamethasone, 80 μM ascorbic acid-2-phosphate and 10 ng ml^−1^ transforming growth factor β3. The 2 × 5 mm scaffolds required 1 ml of differentiation media and the 6 × 5 mm scaffolds required 1.5 ml of differentiation media. The 24-well plate was incubated at 37 °C on a rotating platform, with media changes three times a week, and after 7 days, the media was supplemented with 10 μg ml^−1^ human pancreatic insulin to promote matrix secretion. The constructs were harvested after 35 days for histological and biochemical analysis, or at earlier intervals for qPCR. For each condition tested, two constructs were digested for biochemical analysis, one was sectioned for histology, and two were processed for qPCR.

### Biochemical analysis

Engineered cartilage constructs were pooled, lyophilized and digested at 37 °C overnight and 67 °C for 3 h using 250–1,000 μl of digestion solution containing 2 mg ml^−1^ L-(tosylamido-2-phenyl) ethyl chloromethyl ketone (TCPK)-treated trypsin, 1 mM EDTA, 1 mM iodoacetamide, 0.02 mg ml^−1^ pepstatin A and 50 mM Tris-HCl at pH 7.2. The cartilage digest was then boiled for 15 min at 100 °C, centrifuged for 2 min at 15,000g and then analysed for GAG using a chondroitin sulfate assay or type I or type II collagen using enzyme-linked immunosorbent assays. For full details of digestion and biochemical assays, see Supplementary Methods.

### Histological analysis

Engineered cartilage constructs were fixed overnight using 40 mg ml^−1^ paraformaldehyde in PBS, immersed in 70% ethanol (v/v) for 2 h and then submitted to the Histology Services Unit (University of Bristol). Here, the cartilage was embedded in paraffin wax, and 3 μm sections were taken from the centre of the tissue and affixed to polysine microscope slides (VWR, UK). The cartilage sections were stained using haematoxylin and eosin, safranin O, while immunohistochemistry was used to locate type I collagen and type II collagen. Images were captured using a DMI 3,000 optical microscope (Leica, UK) and image analysis was performed on safranin O sections using Image J (National Institute of Health, USA). For full details of staining and image analysis, see Supplementary Methods.

### RNA extraction and qPCR analysis

Engineered cartilage constructs were washed with PBS and the inner tissue segment was isolated using a 3 mm biopsy punch (Stiefel Laboratories, USA) before RNA extraction using a Total RNA Purification Kit (Norgen Biotek Corp, Canada). RNA concentration was measured using a DU530 spectrophotometer (Beckmann Coulter, USA) and a maximum of 2,000 ng of RNA was used to synthesize 40 μl of cDNA using a PrimeScript RT reagent kit (Takara, Japan) and a MJ Mini thermal cycler (BioRad Laboratories, USA) with a 37 °C and 85 °C annealing and denaturation temperature, respectively. qPCR was performed using a 7500 real-time Applied Biosystems Detection System and 96-well human hypoxia Taqman array plates (Life Technologies, USA, Catalogue Number 4418735) with the cDNA template diluted to 50 ng μl^−1^. For full details of RNA extraction and qPCR analysis, see Supplementary Methods.

## Additional information

**How to cite this article:** Armstrong, J.P.K. *et al*. Artificial membrane-binding proteins stimulate oxygenation of stem cells during engineering of large cartilage tissue. *Nat. Commun*. **6**:7405 doi: 10.1038/ncomms8405 (2015).

## Supplementary Material

Supplementary Information Supplementary Figures 1-24, Supplementary Tables 1-3, Supplementary Methods and Supplementary References
