# Supplementary material for: Artificial membrane-binding proteins stimulate oxygenation of stem cells during engineering of large cartilage tissue
Source: Nat Commun. 2015 Jun 17;6:7405. doi: 10.1038/ncomms8405 (PMC4557285; doi:10.1038/ncomms8405)
Supplement: Supplementary Information — Supplementary Figures 1-24, Supplementary Tables 1-3, Supplementary Methods and Supplementary References [file ncomms8405-s1.pdf]

Supplementary Figures

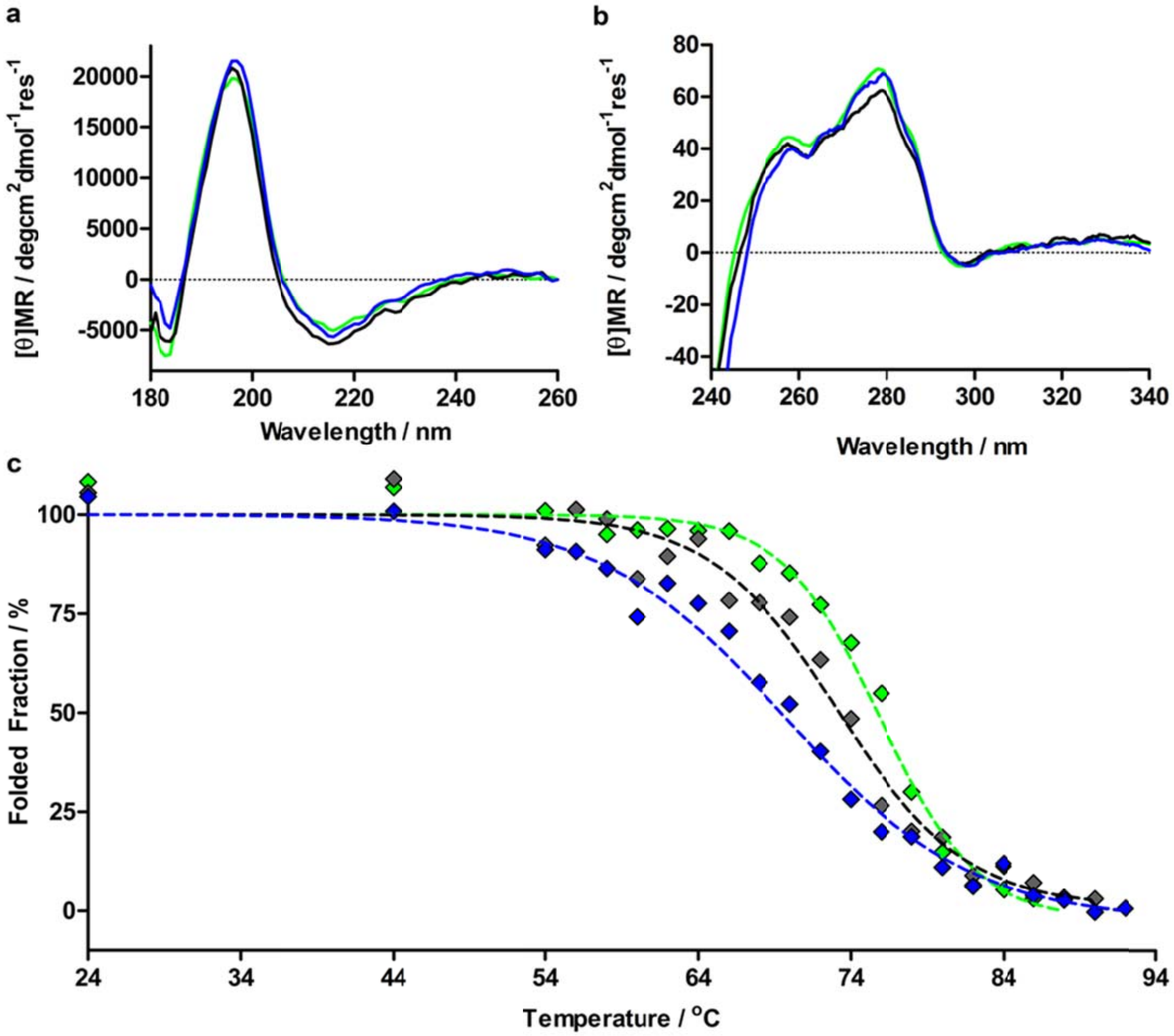

Supplementary Figure 1 Structural characterisation of the eGFP complexes. Synchrotron radiation circular dichroism (SCRD) spectroscopy was used to analyse the structure of eGFP (green trace), [eGFP_C] (black trace) and [eGFP_C][S] (blue trace). (**a**) Far-UV SCRD and (**b**) near-UV SCRD showed superimposed traces indicating retention of native secondary and tertiary structure, respectively. (**c**) The molar ellipticity at 196 nm was measured during thermally-induced denaturation of [eGFP_C] and [eGFP_C][S] (data points), and fitted to sigmoidal curves (dashed lines). The half denaturation temperature of eGFP, [eGFP_C] and [eGFP_C][S] were estimated at 76, 73 and 70°C, respectively.

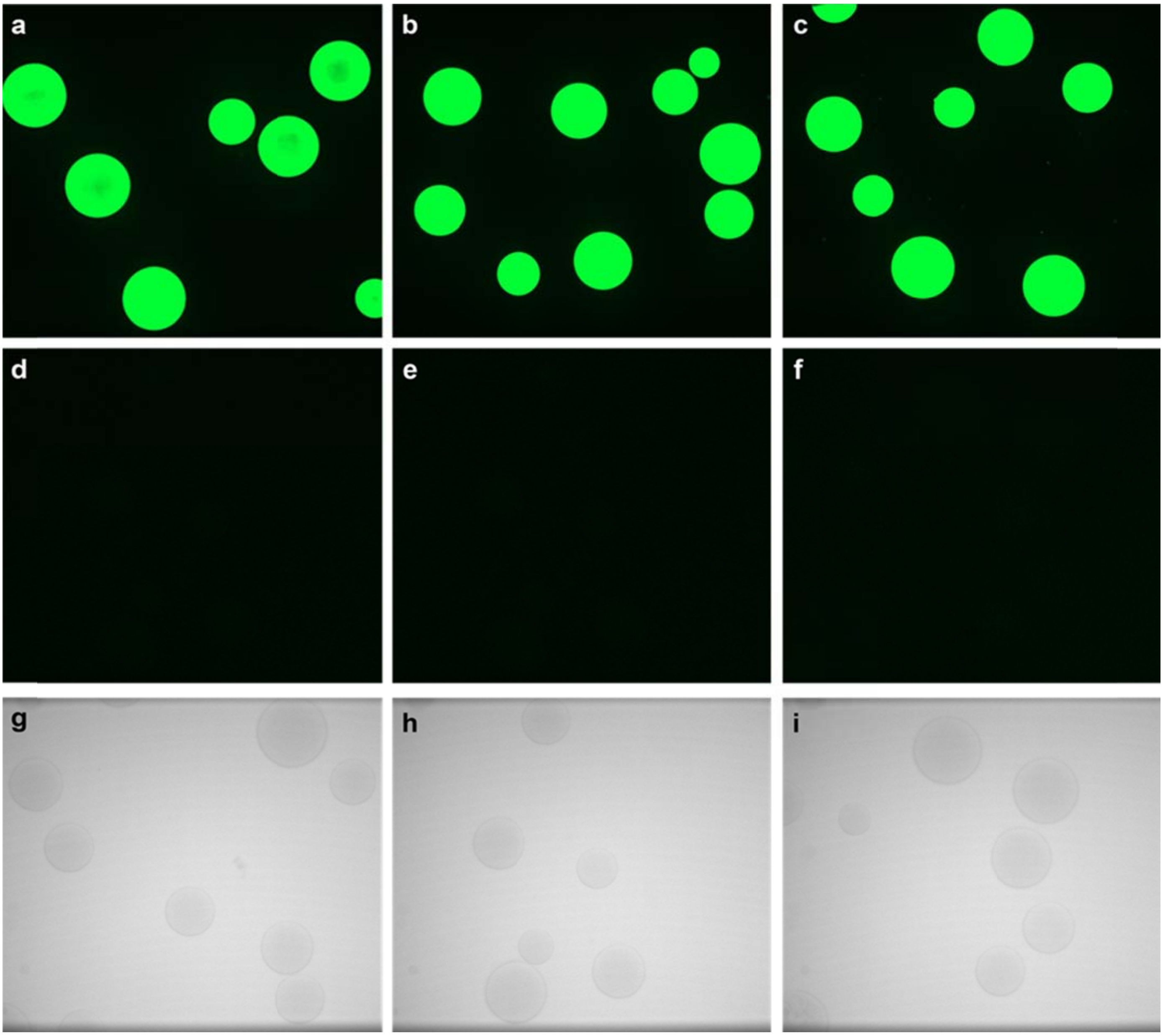

Supplementary Figure 2 eGFP antibody binding study. Confocal microscopy images showing GFP-Trap® beads bound to (**a**) eGFP, (**b**) [eGFP_C] and (**c**) [eGFP_C][S]. No binding was observed when using blocked agarose control beads for either (**d & g**) eGFP, (**e & h**) [eGFP_C] and (**f & i**) [eGFP_C][S], as shown by confocal / bright field microscope images.

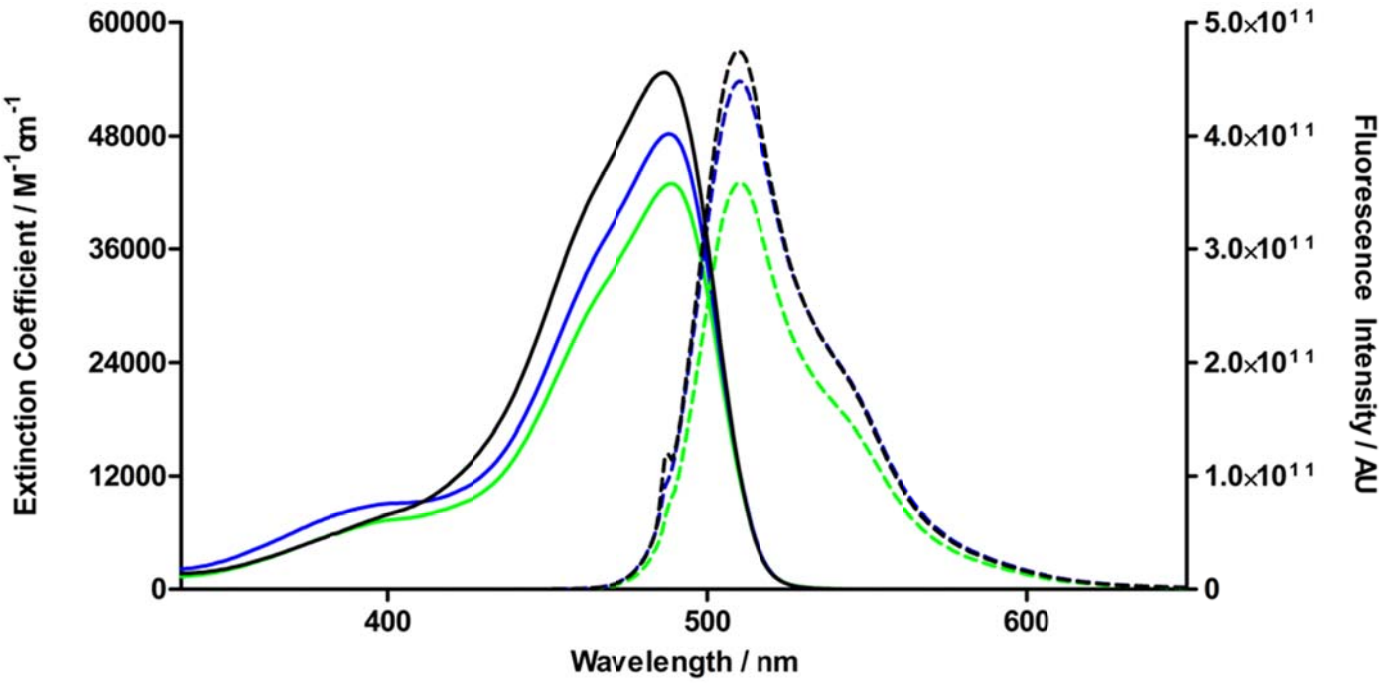

Supplementary Figure 3 Optical properties of the eGFP complexes. UV-visible spectroscopy (solid lines, left y-axis) and fluorescence spectroscopy (dashed lines, right y-axis) showing the optical characteristics of eGFP (green trace), [eGFP_C] (black trace) and [eGFP_C][S] (blue trace). In each case, the primary absorption band was observed at 488 nm, with a corresponding emission peak at 508 nm.

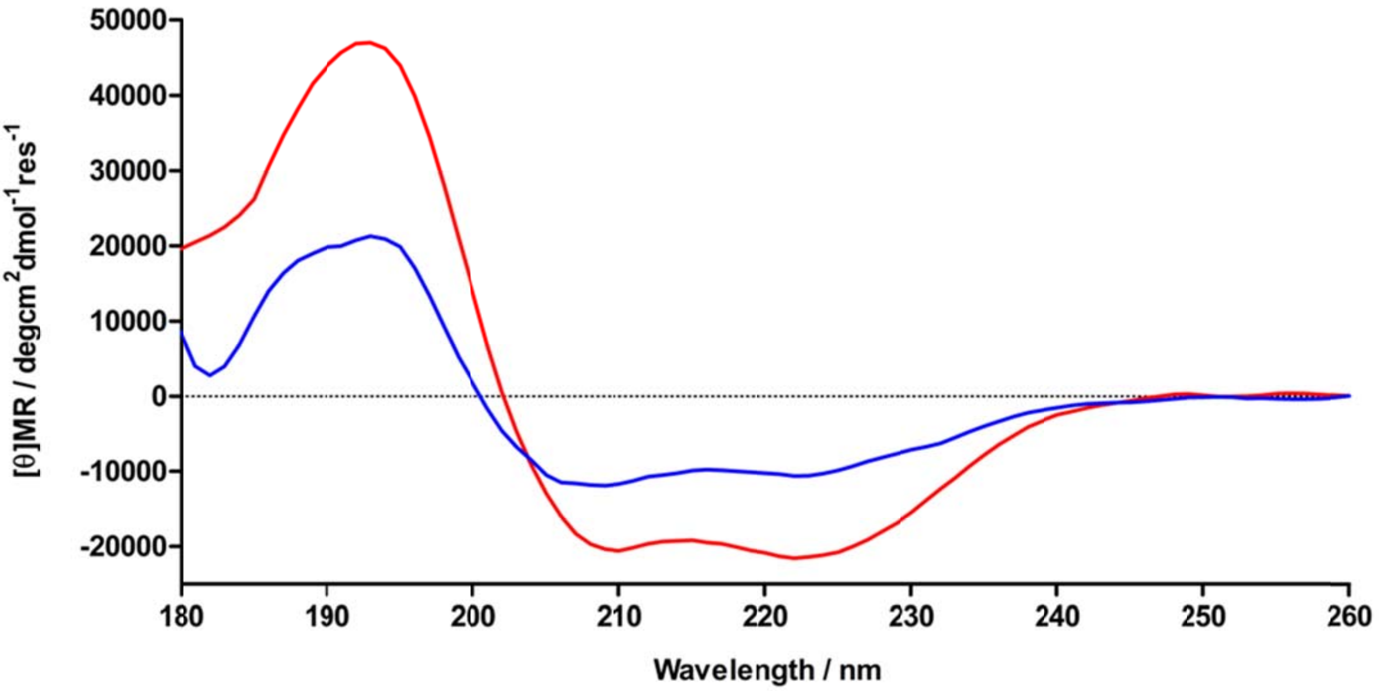

Supplementary Figure 4 Structural characterisation of the [Mb_C][S]. Far-UV SCRD was used to analyse the secondary structure of Mb (red) and [Mb_C][S] (blue). The peaks observed at 208 and 222 nm were present in both samples, indicating that [Mb_C][S] retained the predominantly α-helical secondary structure of native Mb, albeit with reduced circular dichroism signal.

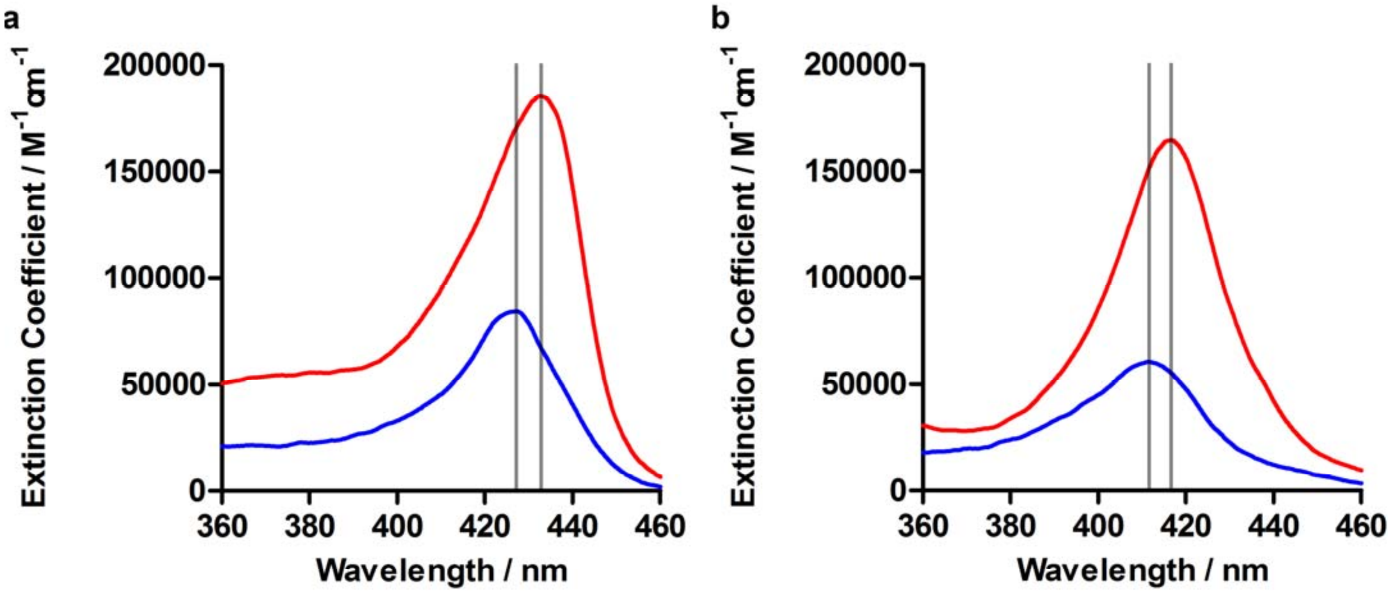

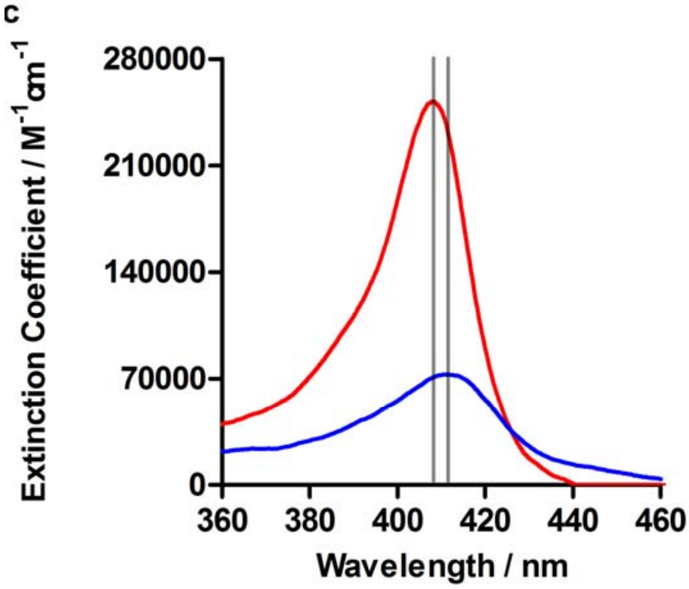

d

Soret Band Wavelength / nm

	Fe (III)	Fe (II) - O_2	Fe (II)
**Mb**	408	416	433
**[Mb_C][S]**	411	414	424

Supplementary Figure 5 Soret band analysis of the Mb complexes. UV-visible spectroscopy was used to study the (a) deoxyferrous, (b) oxyferrous and (c) ferric oxidation states of Mb (red line) and [Mb_C][S] (blue line). The presence of the Soret band in the protein-surfactant complexes indicated retention of the haem porphyrin, however, shifts in wavelength (denoted by grey lines) and reduction in absorbance intensity does suggest conformational changes around the haem.

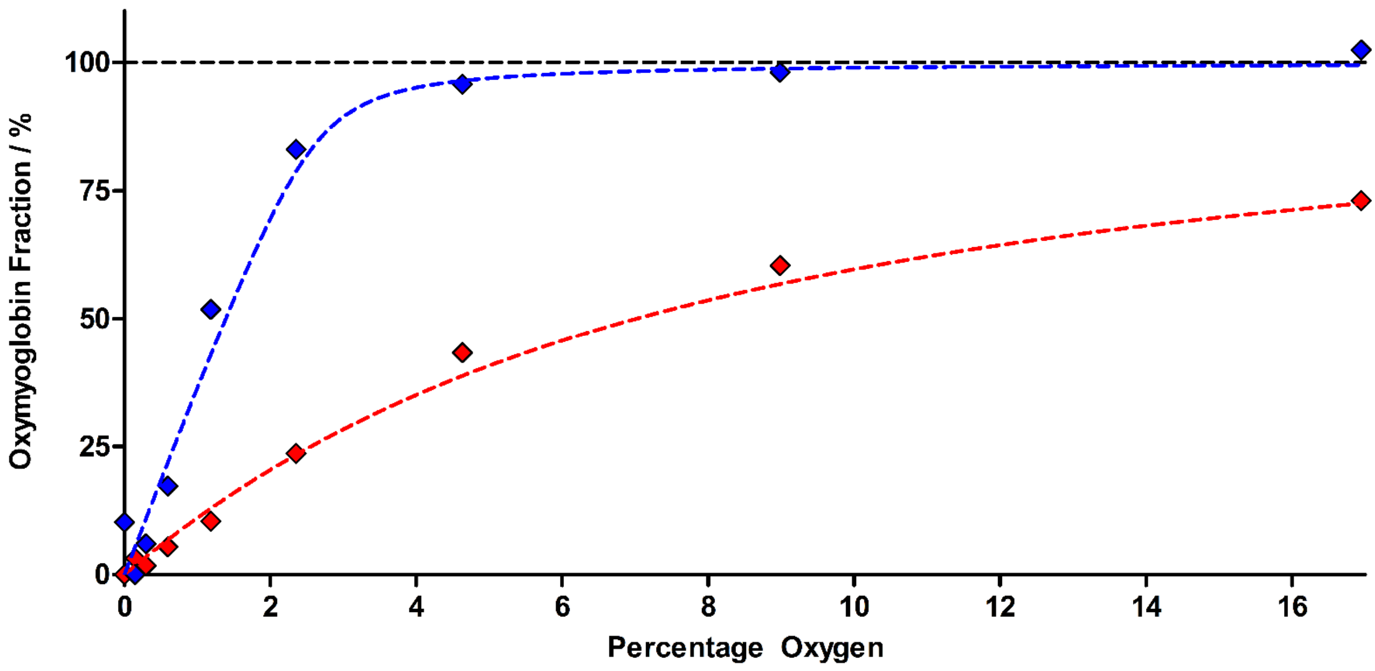

Supplementary Figure 6 Equilibrium oxygen binding by the myoglobin complexes. The Soret peak intensity was used to determine the fraction of oxyferrous haem in solutions of Mb (red data points) and [Mb_C][S] (blue data points), across a range of oxygen concentrations. These were fitted to a function describing the equilibrium between myoglobin and oxygen (dashed lines). [Mb_C][S] exhibited a characteristic, non-linear profile with a higher oxygen affinity than native myoglobin. For spectra of the initial, deoxyferrous solutions and the final, oxyferrous solutions please see Supplementary Figs. 5a and 5b, respectively.

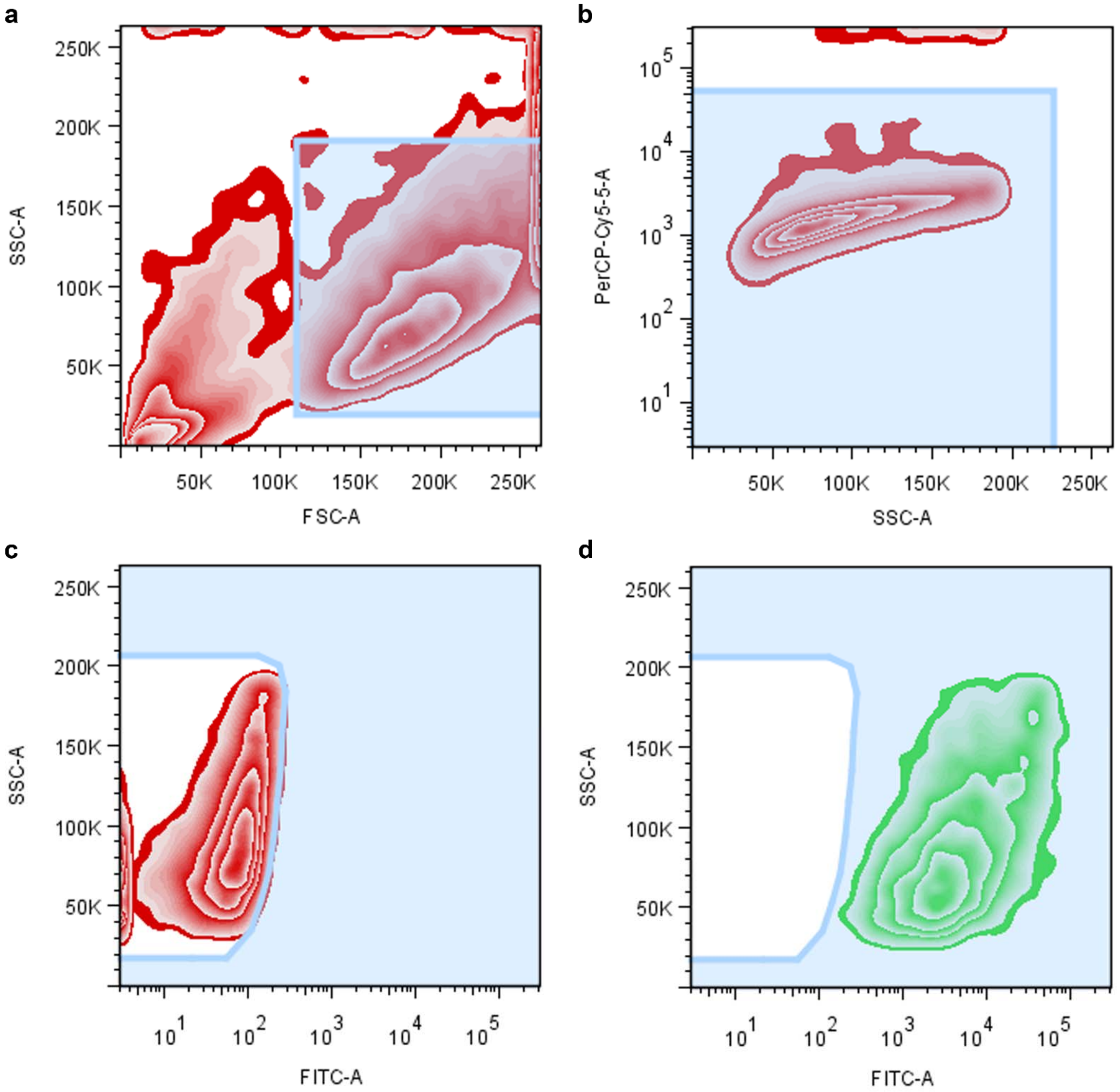

Supplementary Figure 7 Flow cytometry gating parameters**.** The flow cytometry analysis of the cell populations consisted of a three-step gating analysis. (**a**) First, whole cells were identified using a plot of side scatter area (SSC-A) and forward scatter area (FSC-A), parameters that defined cell granularity and size, respectively. (**b**) Secondly, the whole cell population was gated to include only cells with low propidium iodide fluorescence (PerCP-Cy5-5-A), to exclude non-viable cells. (**c**) Finally, unlabelled cells were used to established an eGFP-negative cell population using the green fluorescence channel (FITC-A) (d) When this gating analysis was applied to cells primed with [eGFP_C][S], the mean fluorescence intensity and proportion of labelled cells could be determined.

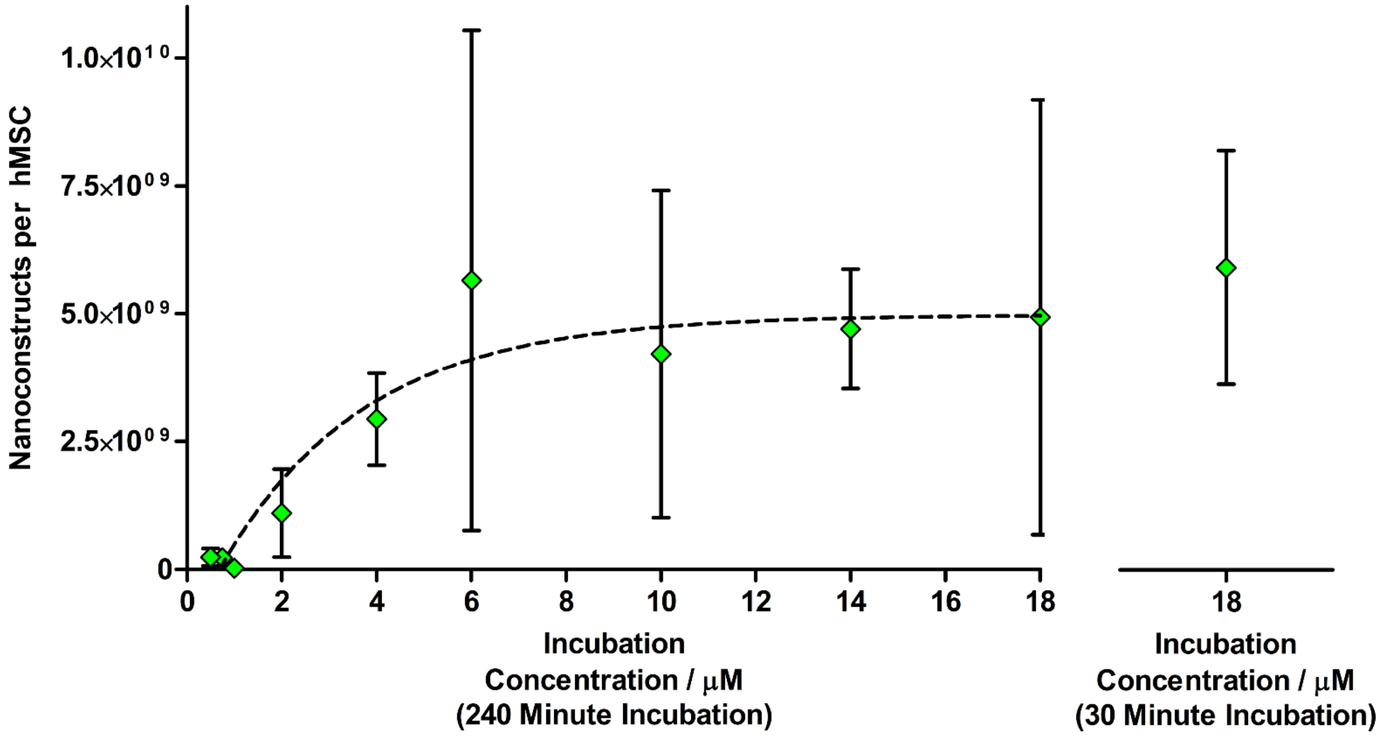

Supplementary Figure 8 Estimating the number of complexes per cell**.** hMSCs were incubated for four hours across a range of [eGFP_C][S] concentrations to reach equilibrium conditions, then fluorescence spectroscopy was used to compare the depleted supernatant to the initial solutions. A reduction in the intensity of the characteristic emission band at 508 nm allowed the number of [eGFP_C][S] molecules per cell to be estimated for each incubation concentration. A plateau was reached at an incubation concentration of around 6 µM, which corresponds to approximately five billion [eGFP_C][S] molecules per cell. A similar saturation level was reached within 30 minutes using the incubation concentration of 18 µM (*c.f.* $5.9 \pm 2.3 \times 10^9$ complexes per cell). The bars represent the mean average and standard deviation calculated based on fluorimetry experiments using supernatant of primed hMSCs from two different patients.

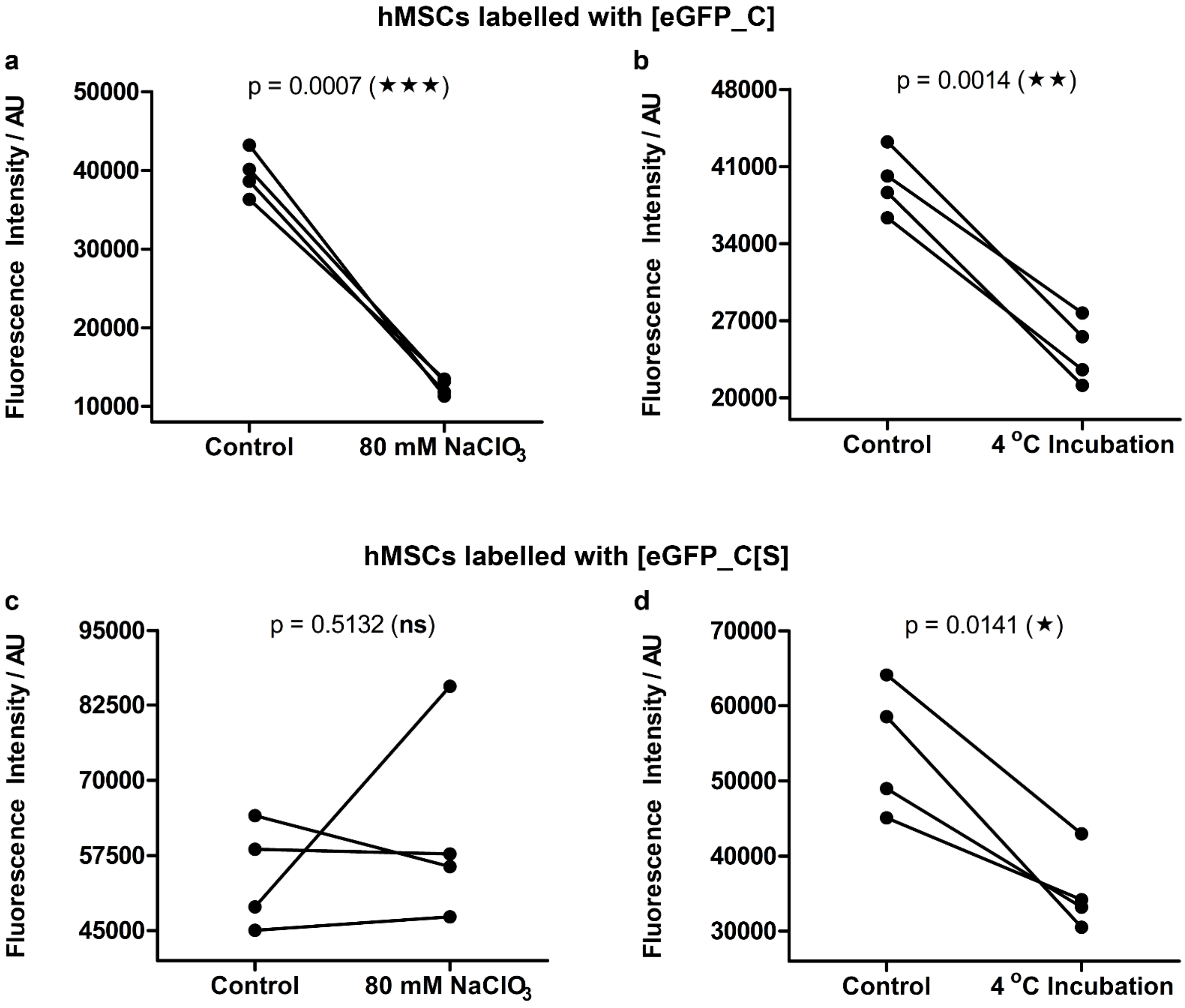

Supplementary Figure 9 Membrane binding mechanisms. Flow cytometry was used to elucidate the cell-protein association mechanisms for [eGFP_C] and [eGFP_C][S]. (**a**) [eGFP_C] showed significantly reduced membrane affinity towards cells cultured in sodium chlorate, which inhibits the synthesis of anionic proteoglycans on the cell membrane, and (**b**) a smaller reduction when the incubation temperature was reduced. (**c**) In contrast, the labelling efficiency of [eGFP_C][S] was not affected by sodium chlorate supplementation, while (**d**) reducing the temperature again decreased membrane association. Taken together, these results suggest distinct membrane-binding mechanisms, with the cationised protein predominantly mediated by electrostatic interactions, whereas the eGFP complexes associates in a non-electrostatic fashion, dependent upon membrane fluidity. The data was collected using cells from four paired patients, and comparison of differences was tested using a two-tailed paired t-test with a p-value of less than 0.05 considered significant.

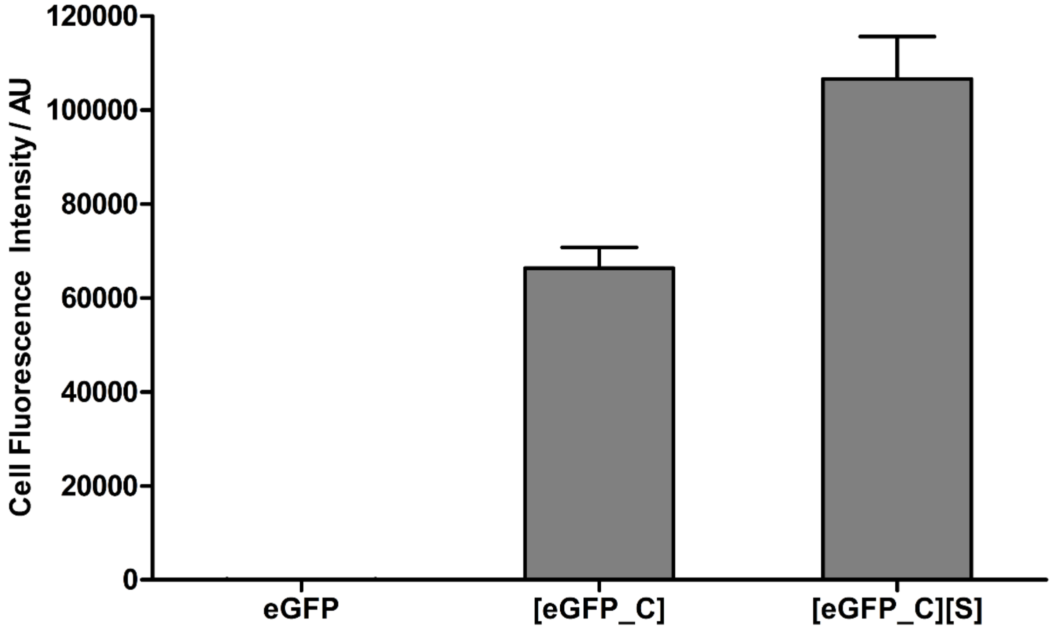

Supplementary Figure 10 Membrane affinity of eGFP complexes. Flow cytometry was used to analyse the fluorescence of the primed cells, and thus the membrane affinity of [eGFP_C] and [eGFP_C][S]. The cells primed with native protein exhibited low fluorescence intensity, with only 0.7 ± 0.2% of the population classed as eGFP-positive. Both [eGFP_C] and [eGFP_C][S] labelled the entire cell population, however, the cells primed with protein-surfactant complexes exhibited a higher fluorescence intensity than the cells primed with [eGFP_C]. The bars represent the mean average and standard deviation calculated based on flow cytometry measurements using hMSCs from four different patients.

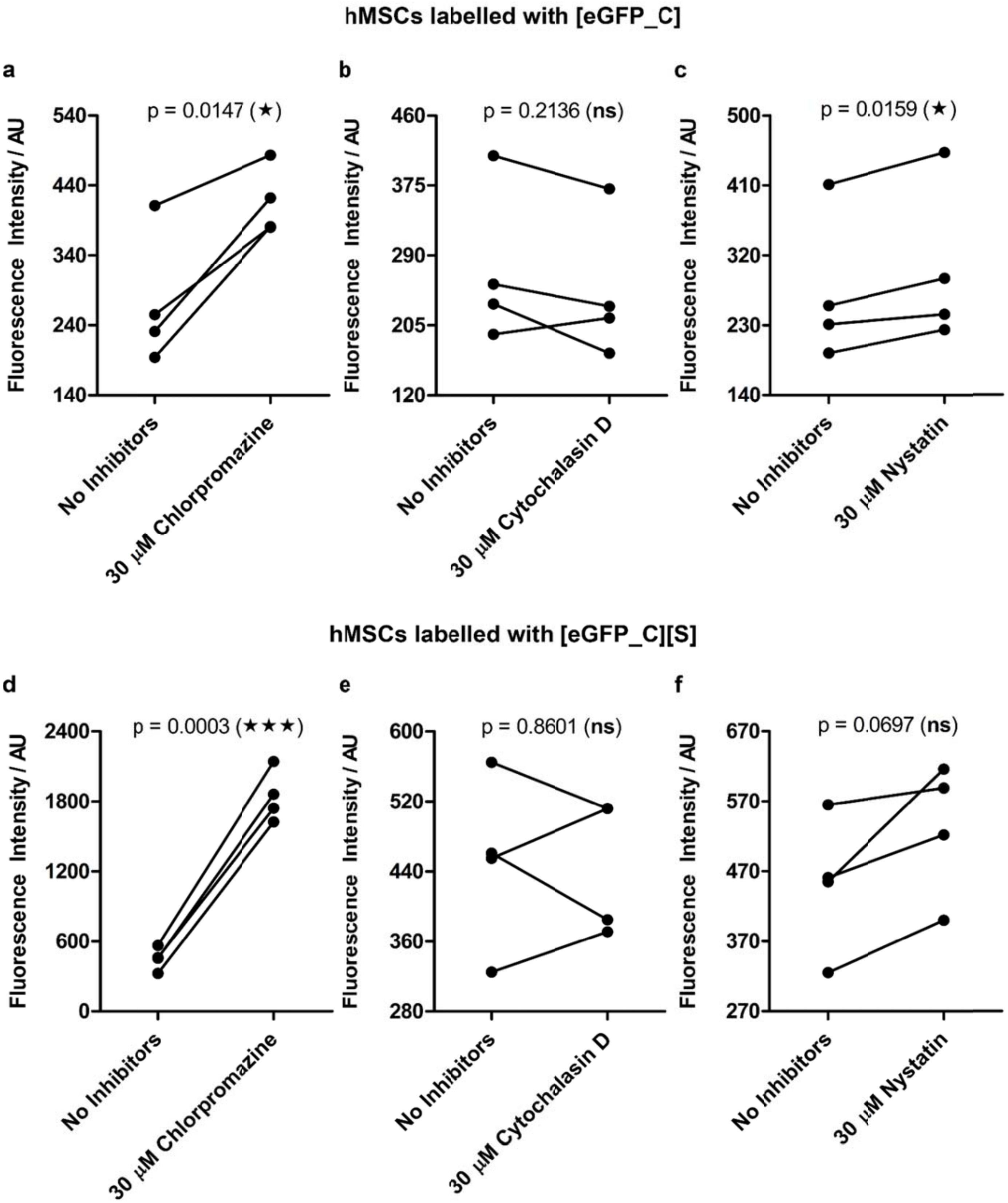

Supplementary Figure 11 Mechanisms of endocytosis. Flow cytometry was used to elucidate the mechanism of endocytosis for membrane-bound [eGFP_C] and [eGFP_C][S]. (**a & d**) Supplementing the culture medium with chlorpromazine significantly increased the persistence time of both [eGFP_C] and [eGFP_C][S]. Smaller increases were observed with (**b & e**) nystatin, while (**c & f**) cytochalasin D had no significant effect. This suggests that the membrane-bound protein is predominantly processed *via* clathrin-mediated endocytosis. The data was collected using cells from four paired patients, and comparison of differences was tested using a two-tailed paired t-test with a p-value of less than 0.05 considered

significant.

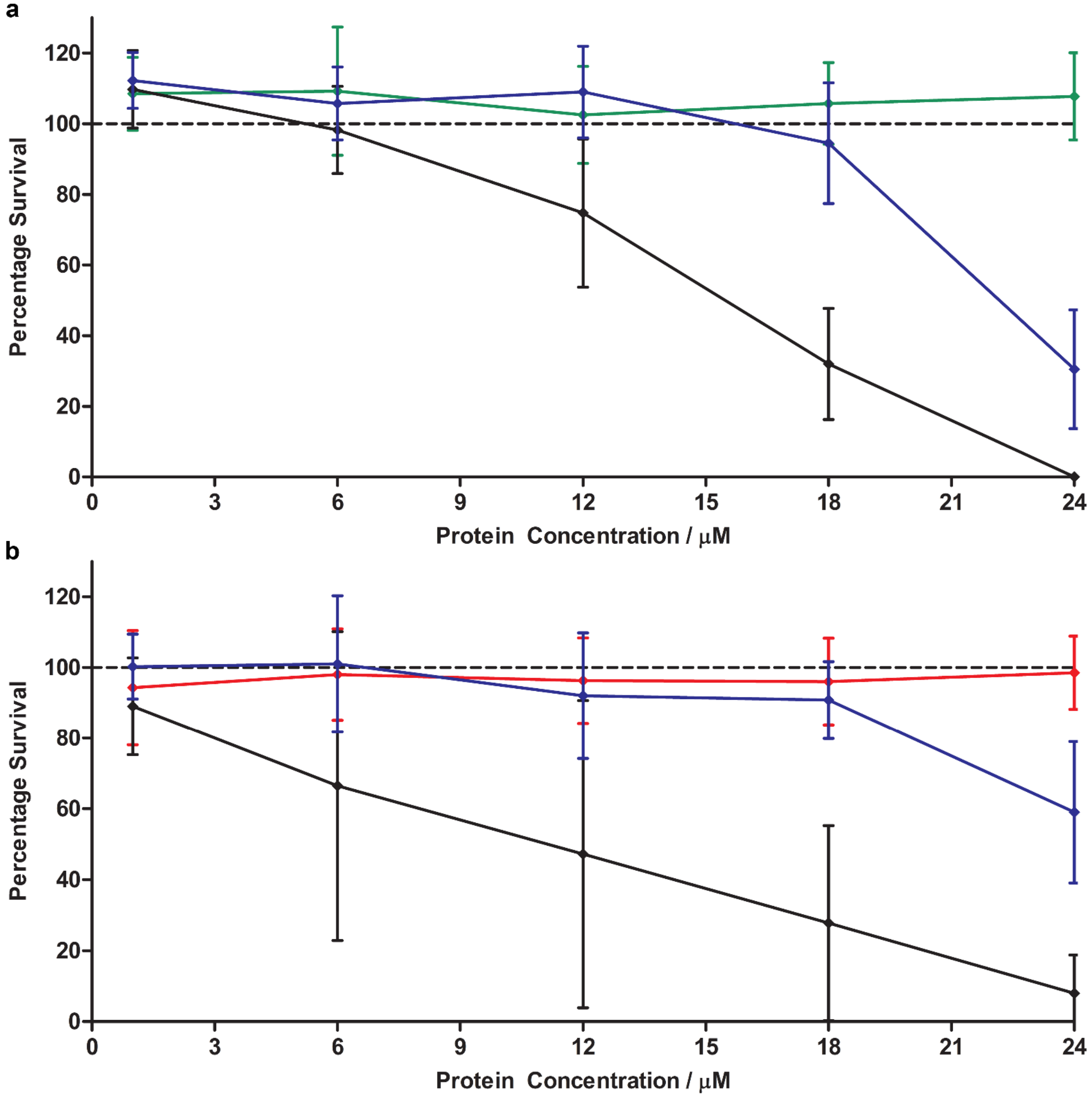

Supplementary Figure 12 Cell viability assay. A cytotoxicity assessment was performed using (3-(4,5-dimethylthiazol-2-yl)-5-(3-carboxymethoxyphenyl)-2-(4-sulfophenyl)-2H-tetrazolium) (MTS) on hMSCs primed for 30 minutes with (**a**) eGFP (green trace), [eGFP_C] (black trace) and [eGFP][S] (blue trace) and (**b**) Mb (red trace), [Mb_C] (black trace) and [Mb_C][S] (blue trace). The complexes exhibited no cytotoxicity at the working concentration of 18 μM, similar to the native proteins that do not associate with the cell. The cationised proteins, however, are cytotoxic at high concentrations, this difference can perhaps be rationalised by the observed differences in membrane association.

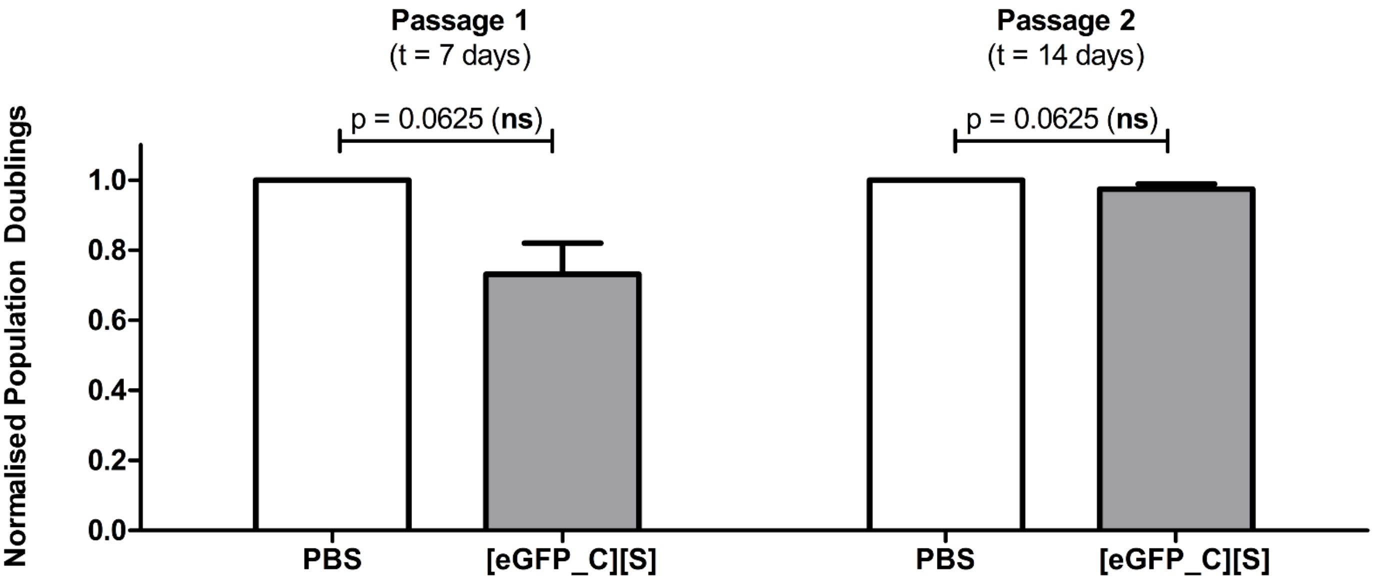

Supplementary Figure 13 Cell proliferation assay. Cell counts were performed on culture-expanded hMSCs primed with [eGFP_C][S], and compared to unlabelled cells. The primed cells retained their self-renewal capability, albeit with a slightly reduced proliferation rate (73%) compared to the unlabelled cells, for the first seven days in culture. After 14 days in culture, the primed hMSCs were close to full proliferative capacity (97%), which is concurrent with clearance of the membrane-bound complexes, indicating no long-term detrimental effects on cell self-renewal. The bars represent the mean average and standard deviation calculated based on cell counts of hMSCs from five separate patients. Comparison of differences was tested using a Wilcoxon paired analysis, with two-tailed p-value of less than 0.05 considered significant.

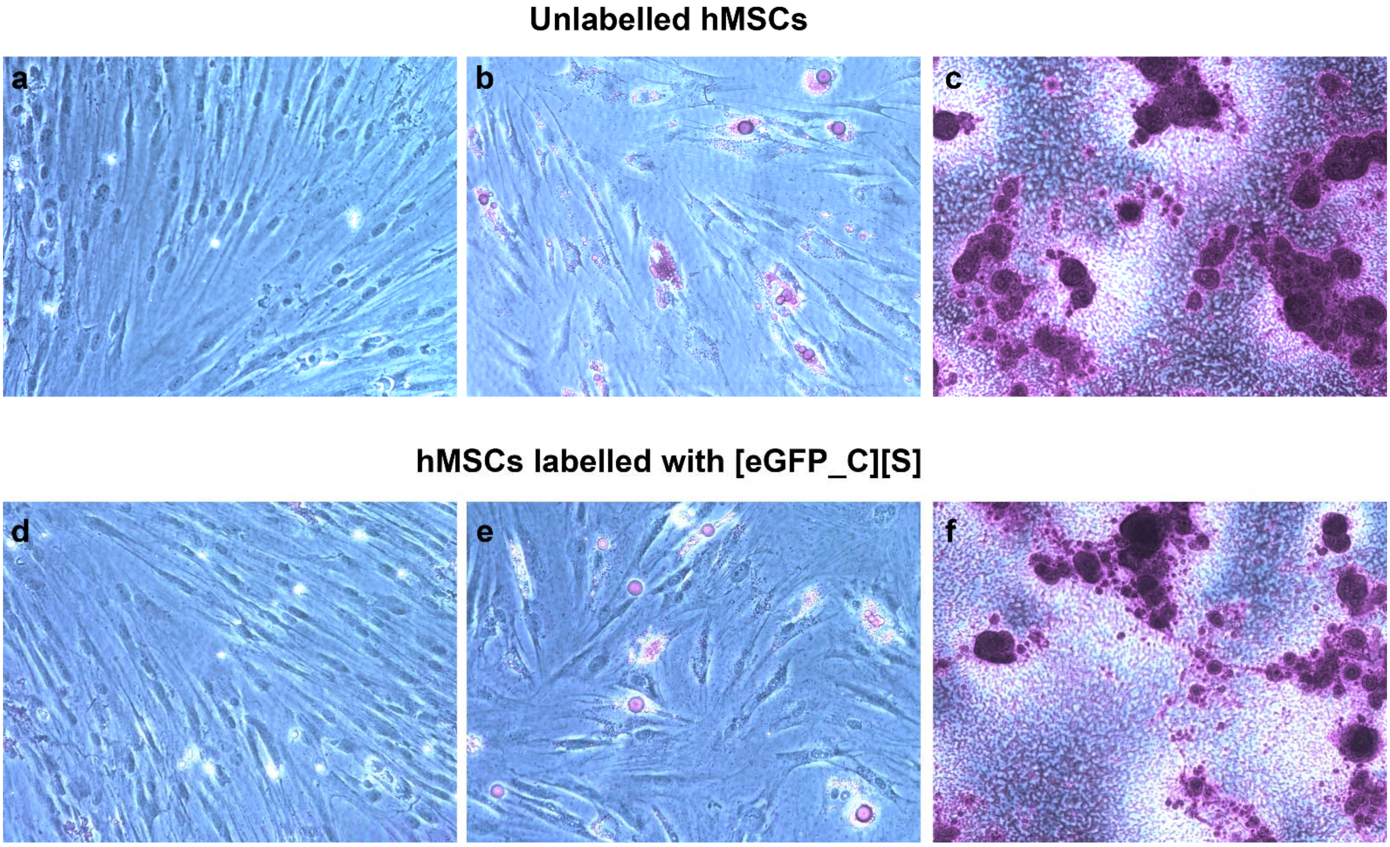

Supplementary Figure 14 Monolayer differentiation studies. Osteogenesis and adipogenesis was carried out using hMSCs primed with [eGFP_C][S], and compared to unlabelled hMSCs. (**a & d**) The undifferentiated hMSCs were indistinguishable and, upon differentiation, both sets of cells produced (**b & e**) adipocytes with lipid vacuoles visible upon oil red staining and (**c & f**) osteoblasts with calcium deposits that were stained using alizarin red.

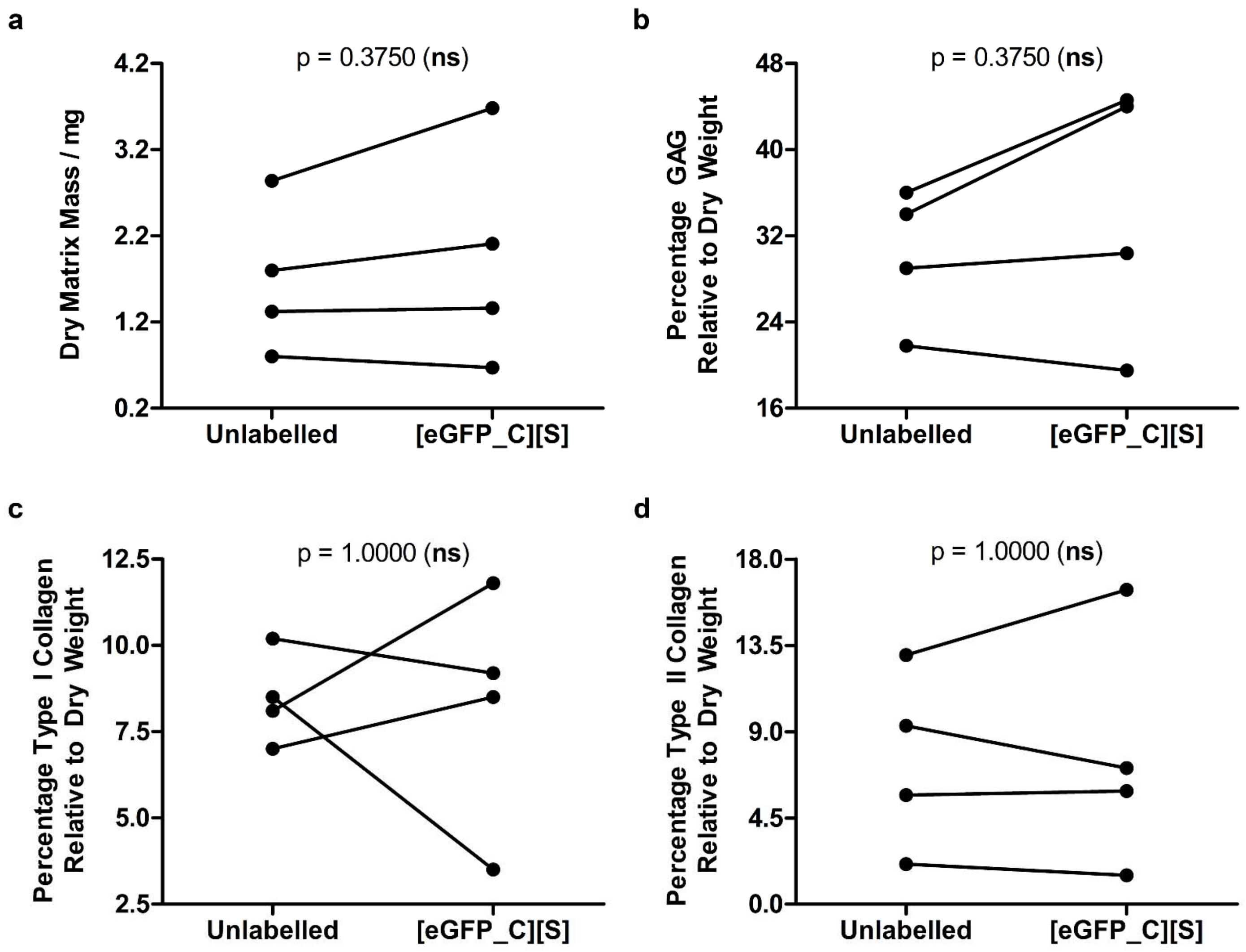

Supplementary Figure 15 Chondrogenesis study. Cartilage tissue engineering was carried out using hMSCs primed with [eGFP_C][S], and compared to a control tissue engineered using unlabelled hMSCs. Biochemical analysis of the digested cartilage showed similar levels of (**a**) matrix deposition, (**b**) glycosaminoglycan content and (**c & d**) collagen production. This is evidence that primed hMSCs retain the ability to undergo chondrogenesis and form cartilage tissue. Four separate patients were used in the study and comparison of differences was tested using Wilcoxon non-parametric paired analysis, with two-tailed p-value of less than 0.05 considered significant.

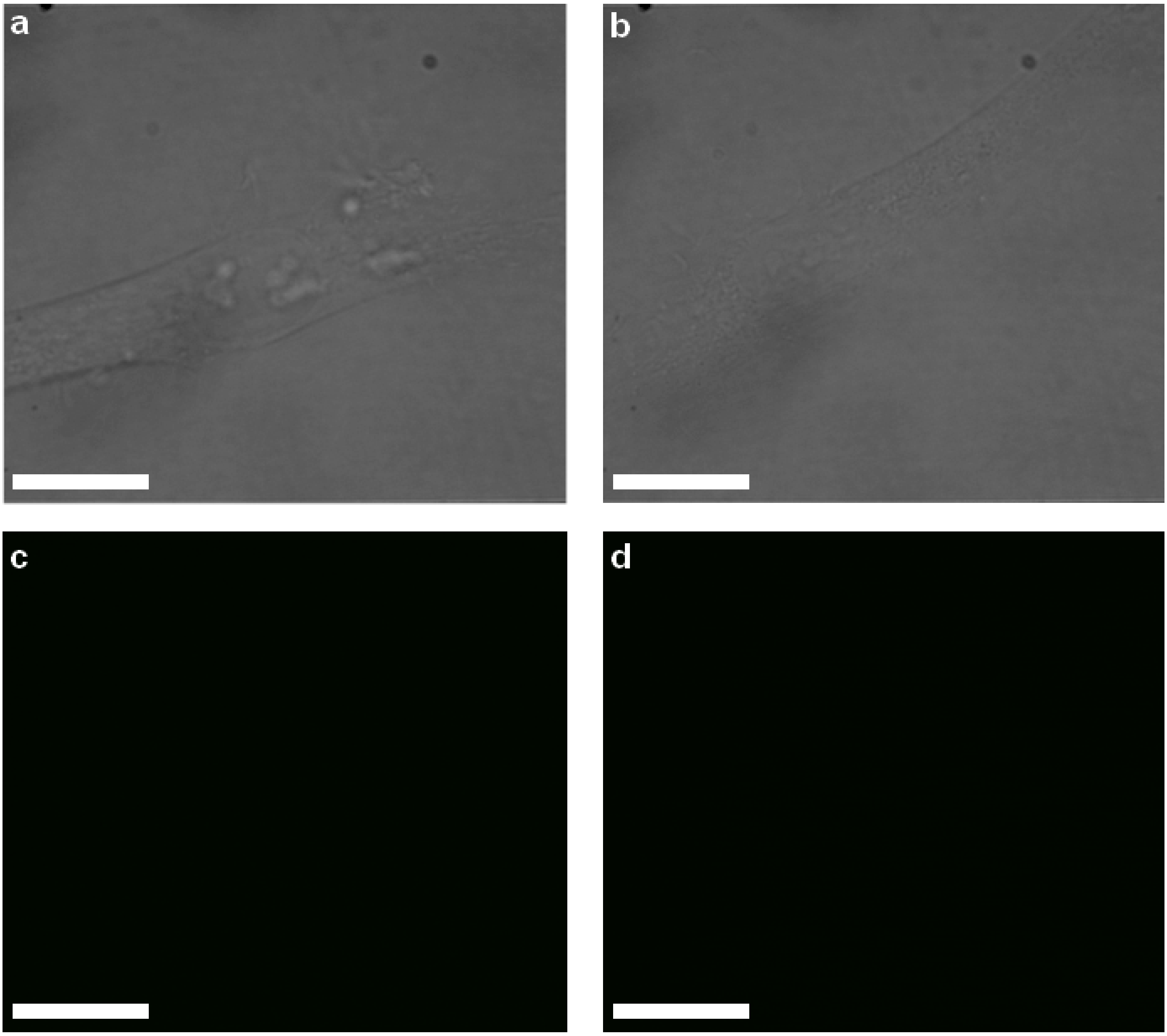

Supplementary Figure 16 Live-cell confocal microscopy controls. (**a-b**) Bright field and corresponding (**c-d**) confocal fluorescence microscopy images depicting a lack of fluorescence on unlabelled hMSCs. These images confirm that the fluorescence observed at the membrane of [eGFP_C][S]-primed hMSCs was not due to autofluorescence. The scale bars in all images represent 25 μm.

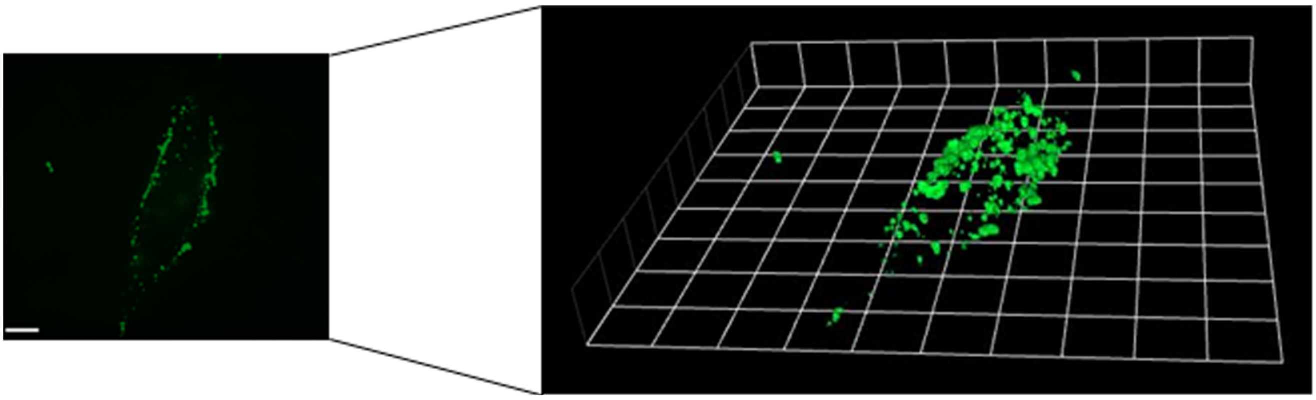

Supplementary Figure 17 Three-dimensional image reconstruction. This analysis demonstrated that [eGFP_C][S] is not homogenously spread across the cell membrane; rather it appears to diffuse laterally into discrete clusters. Given that clathrin-mediated endocytosis is the principal cellular mechanism for internalising [eGFP_C][S], it is likely that the protein complexes are accumulated within clathrin-coated pits. The scale bar in the two-dimensional image is equal to 17 μm.

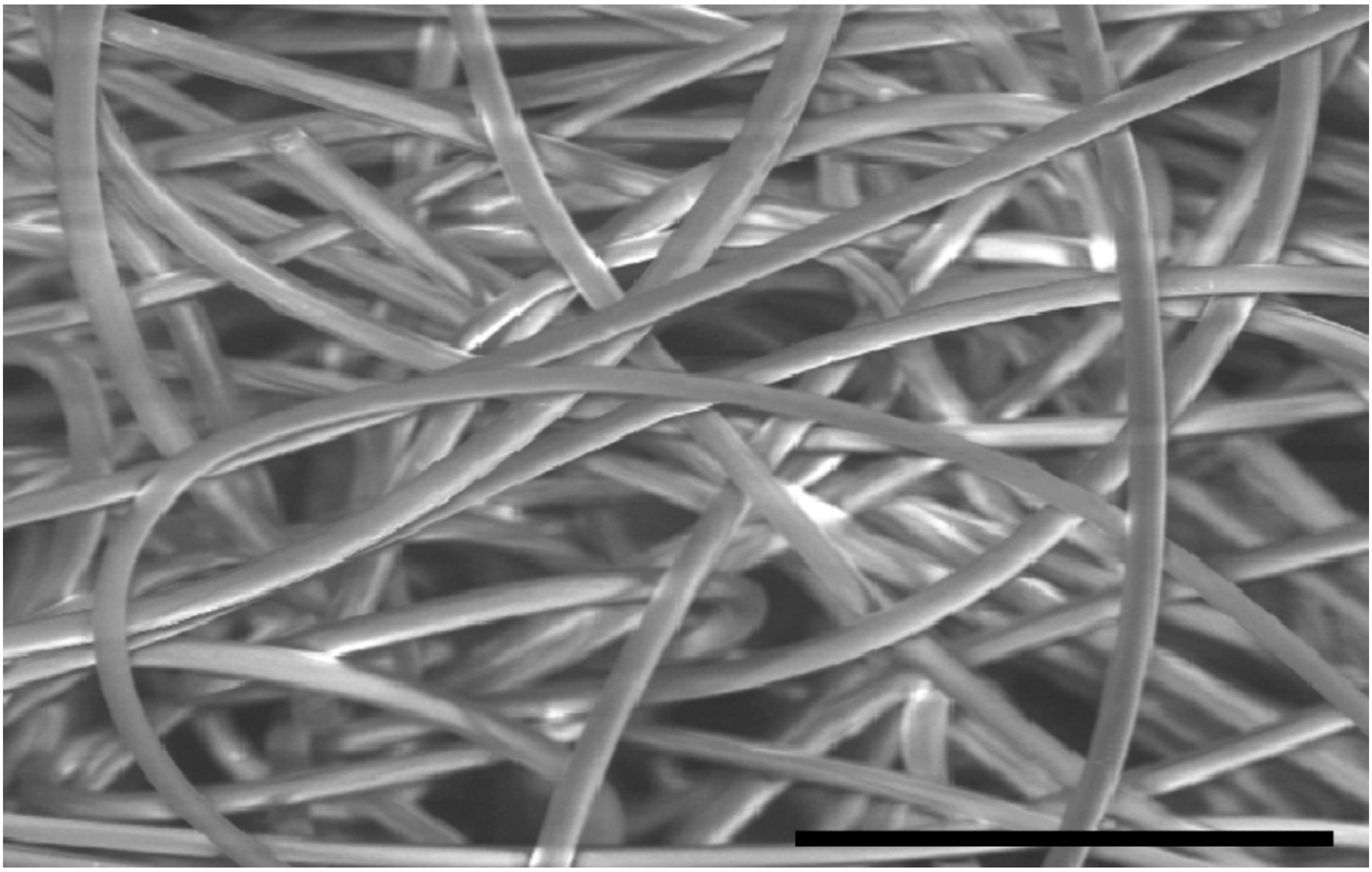

Supplementary Figure 18 Scanning electron microscopy of PGA scaffolds. A scanning electron microscope image of the Biofelt© PGA scaffolds used for cartilage tissue engineering provided by Biomedical Structures. The scale bar corresponds to 200 μm.

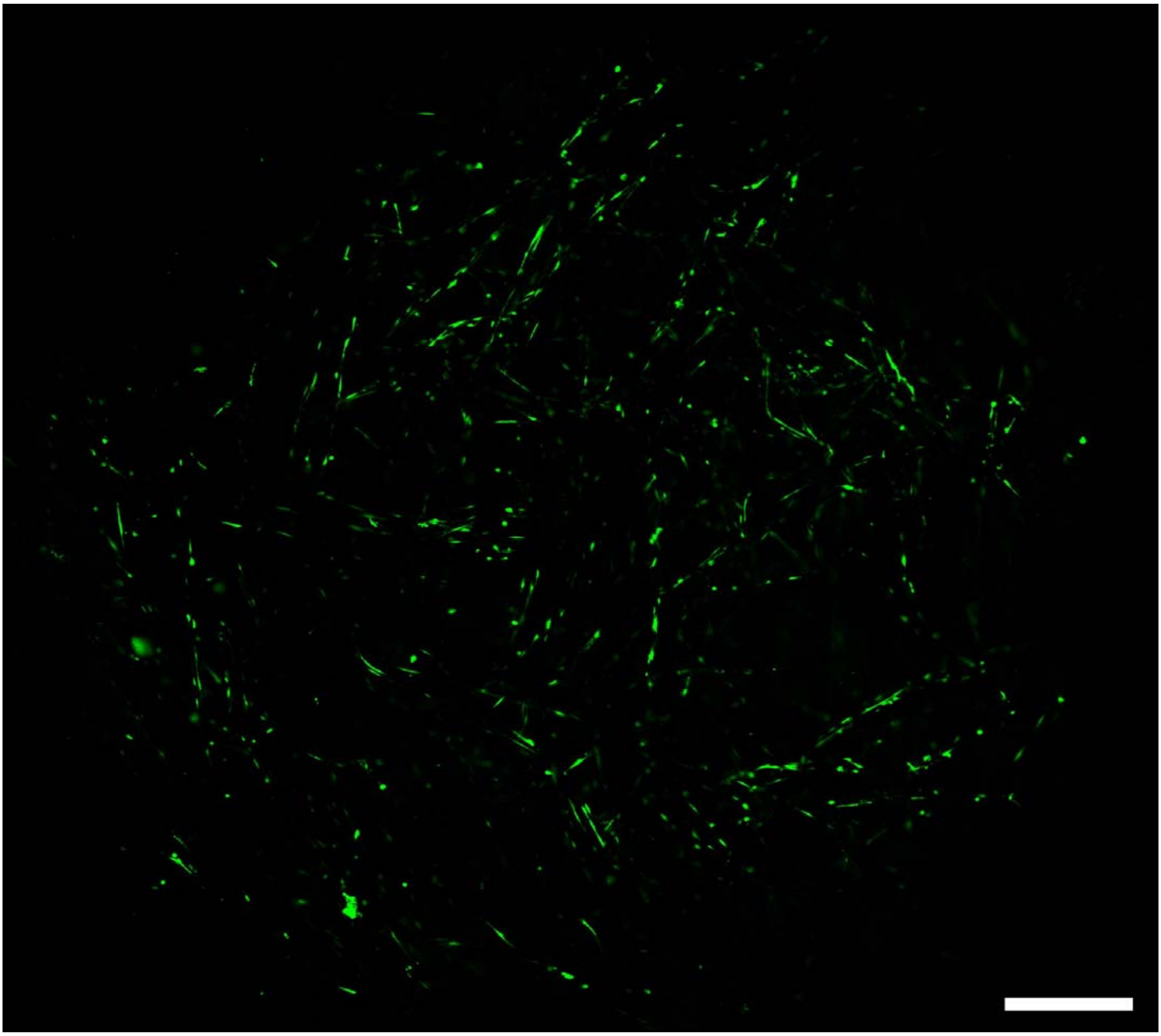

Supplementary Figure 19 Imaging cell distribution in a seeded scaffold. A live-cell confocal fluorescence microscopy image of a PGA scaffold, 24 hours after seeding with calcein-stained hMSCs. The image was taken at a focal distance of approximately half the way through the scaffold and shows a cross-sectional distribution of hMSCs, with a similar number of cells present at the edge and the centre of the scaffold. The scale bar is equivalent to 1 mm.

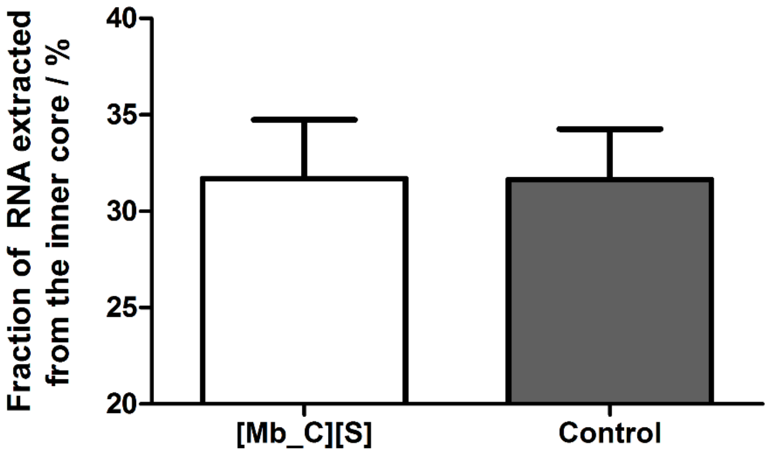

Supplementary Figure 20 Comparison of RNA in seeded scaffolds. The fraction of RNA extracted from the inner core of scaffolds seeded with unlabelled hMSCs (control) or hMSCs primed with 18 μM [Mb_C][S]. The inner core was taken using a 3 mm diameter biopsy punch and represents 36% of the total scaffold volume. For both groups, the inner core contained 32 ± 3% of the RNA, which suggests a reasonably even loading with only a marginally lower cell density measured within the centre of the scaffold.

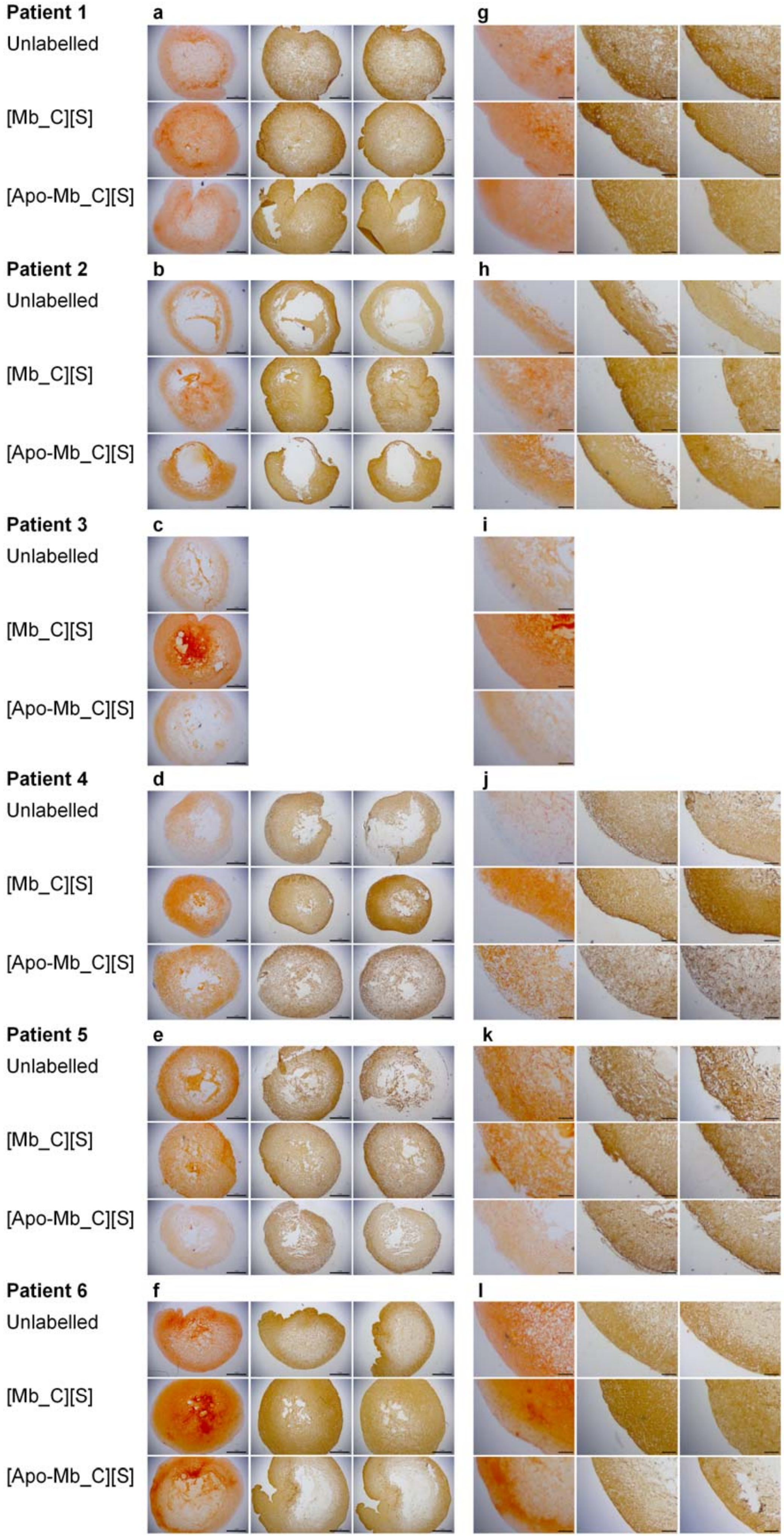
Patient 1
a
g
Unlabelled
[Mb_C][S]
[Apo-Mb_C][S]
Patient 2
b
h
Unlabelled
[Mb_C][S]
[Apo-Mb_C][S]
Patient 3
c
i
Unlabelled
[Mb_C][S]
[Apo-Mb_C][S]
Patient 4
d
j
Unlabelled
[Mb_C][S]
[Apo-Mb_C][S]
Patient 5
e
k
Unlabelled
[Mb_C][S]
[Apo-Mb_C][S]
Patient 6
f
l
Unlabelled
[Mb_C][S]
[Apo-Mb_C][S]

Supplementary Figure 21 Histological analysis of engineered cartilage. Sections taken from the centre of cartilage engineered from unlabelled hMSCs, and hMSCs labelled with [Mb_C][S] and [apo-Mb_C][S] were stained (from left to right) for glycosaminoglycan, type I collagen and type II collagen. Generally, cartilage engineered using hMSCs primed with [Mb_C][S] exhibited superior tissue distribution, compared to the tissue grown with unlabelled hMSCs or hMSCs primed with [apo-Mb_C][S]. All images were taken with the same exposure and gain settings with the scale bar is equivalent to 1 mm (**a-f**) or 250 µm (**g-l**).

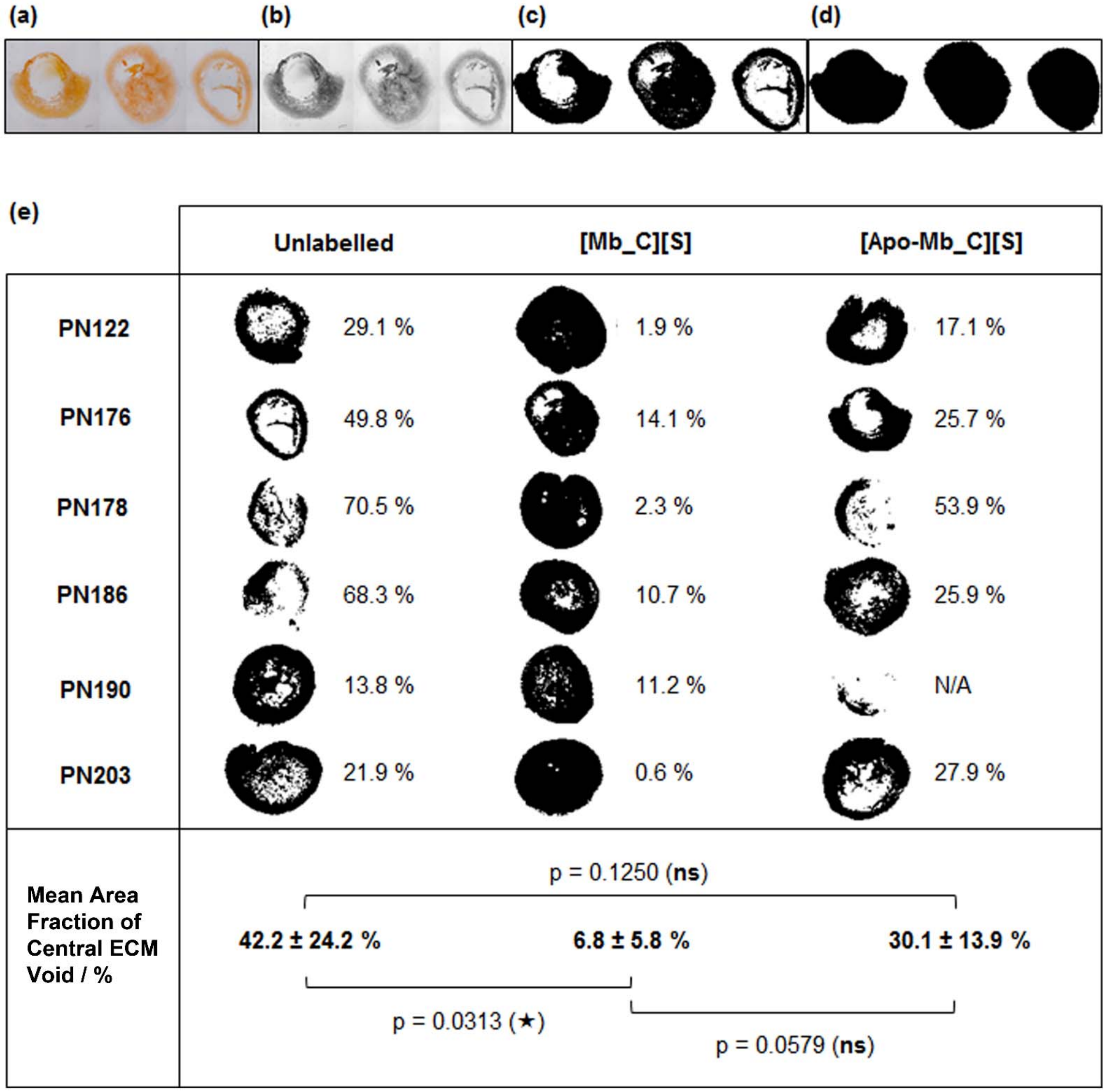

Supplementary Figure 22 Image analysis of cartilage sections. Pixel counting was used as a semi-quantitative measure of the tissue distribution in cartilage engineered using unlabelled hMSCs (control) as well as hMSCs primed with [Mb_C][S] or [apo-Mb_C][S]. Images of safranin O staining were (**a**) collated, (**b**) converted to 16 bit, (**c**) put under mean threshold analysis with outer debris cleared and (**d**) filled in. The threshold images from (**c**) and (**d**) allowed individual particle analysis, and the ratio of the two pixel counts allowed an estimation of (**e**) the percentage necrosis. Pre-treatment with [Mb_C][S] produced cartilage with a significantly reduced void, when compared to the unlabelled cells, whereas [apo-Mb_C][S] had no significant effect. The mean average and standard deviation were calculated based on the tissue engineering using hMSCs from six paired patients, with one section in the [apo-Mb_C][S] set excluded, as no reliable measurement could be made. Comparison of differences was tested using a Wilcoxon non-parametric paired analysis, with a two-tailed p-value of less than 0.05 considered significant.

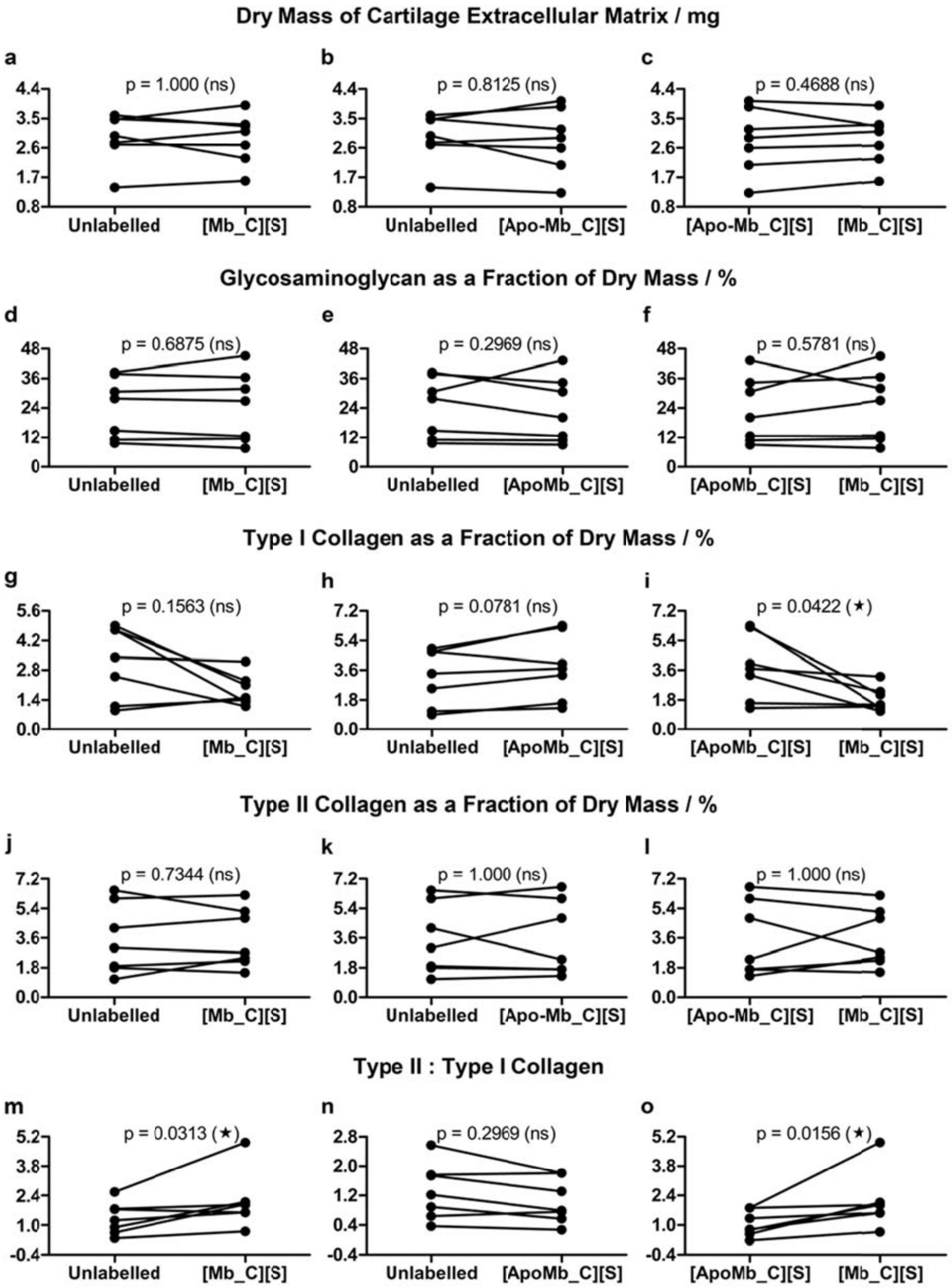

Supplementary Figure 23 Biochemical analysis of engineered cartilage. Cartilage engineered using hMSCs primed with [Mb_C][S] or [apo-Mb_C][S] were digested and compared to a control grown using unlabelled hMSCs. (**a-c**) The dry mass was measured and used to normalise the amount of (**d-f**) glycosaminoglycan, (**g-i**) type I collagen and (**j-l**) type II collagen within each cartilage construct. (**m-o**) The hMSCs primed with [Mb_C][S] produced cartilage with an approximately two-fold increase in the ratio of type II : type I collagen, an indicator that [Mb_C][S] treatment can improve the quality of cartilage tissue engineering. Seven separate patients were used in the study and comparison of differences was tested using Wilcoxon non-parametric paired analysis, with two-tailed p-value of less than 0.05 considered significant and denoted with an asterisk.

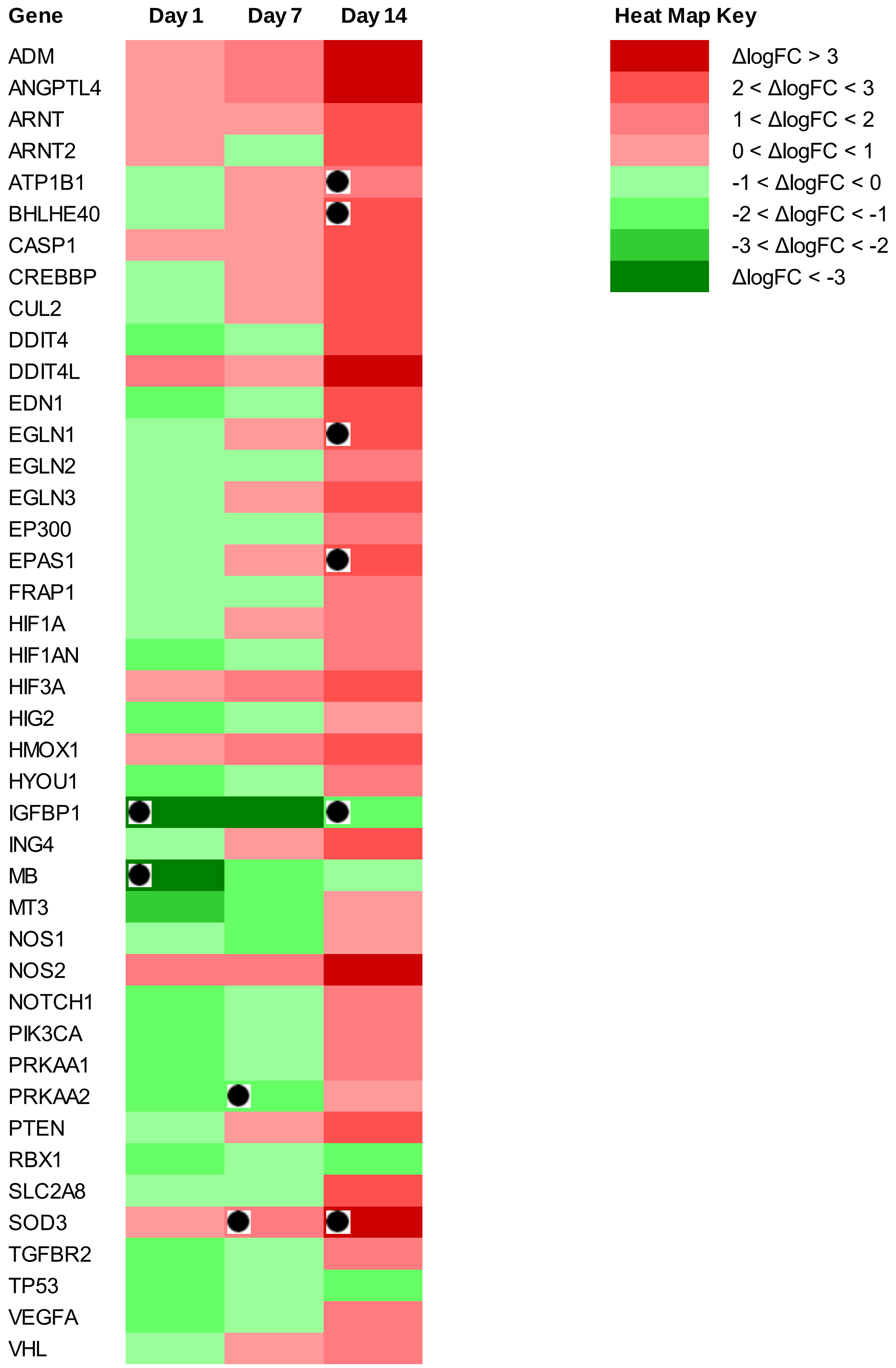

Supplementary Figure 24 Gene expression analysis. The expression of hypoxia markers was compared in cells isolated from the central portion of cartilage engineered using either unlabelled hMSCs or hMSCs primed with 18 μM [Mb_C][S1783]. The fold change (FC) was measured between the two groups at each time point, with a decrease in logFC (green) denoting down-regulation of hypoxia markers and an increase in logFC (red) indicating up-regulation. Three separate patients were used in this study and comparison of differences was tested using a Student t-test analysis, with two-tailed p-value of less than 0.05 considered significant and denoted with a black circle.

Supplementary Tables

Supplementary Table 1 Mass measurements of cationised proteins**.** Matrix-assisted laser desorption/ionization (MALDI) mass spectrometry was used to calculate the mass of the native and cationised proteins to establish the efficiency of the cationisation reaction. The discrepancy in mass corresponds to the covalent addition of approximately 20 and 13 DMPA molecules for eGFP and Mb, respectively.

	**m/z**
**eGFP**	30514
**[eGFP_C]**	32524
**Mb**	16976
**[Mb_C]**	18091

Supplementary Table 2 Zeta potential measurements of the protein-surfactant complexes. Dynamic light scattering was used to measure the zeta potential for the native and cationised proteins. For both eGFP and Mb, cationisation increased the positive charge of the protein, while the surfactant addition neutralised the surface charge through ion pair formation.

	**ζ / mV**
**eGFP**	- 16.8
**[eGFP_C]**	+ 12.6
**[eGFP_C][S]**	+ 2.3
**Mb**	- 11.7
**[Mb_C]**	+ 9.8
**[Mb_C][S]**	- 1.1

Supplementary Table 3 Particle sizing of the protein-surfactant complexes**.** Dynamic light scattering was used to estimate the mean hydrodynamic diameter for the native and modified proteins. For both eGFP and Mb, surfactant addition increased the hydrodynamic diameter of the protein. This is consistent with a compact surfactant corona of around 1-2 nm thickness surrounding the cationised protein.

	**d[H] / nm**	**PDI**
**eGFP**	4.3	0.12
**[eGFP_C]**	4.6	0.13
**[eGFP_C][S]**	8.9	0.33
**Mb**	3.1	0.03
**[Mb_C]**	3.0	0.07
**[Mb_C][S]**	5.7	0.04

Supplementary Methods

Expression and purification of eGFP

BL21 competent *Escherichia coli* (*E. coli*), transformed with the plasmid vector pET45b(+) (Novagen, Germany), was used as an expression host for N-terminal polyhistidine tagged eGFP. The bacteria were cultivated in a Lysogeny Broth (LB) medium, consisting of 10 g L^{-1} of bacto-tryptone (BD Biosciences, USA), 5 gL^{-1} of bacto-yeast extract (BD Biosciences, USA) and 10 g L^{-1} of sodium chloride (Fisher Scientific, UK). An autoclaved 10 mL aliquot of LB media was treated with 10 µL of 50 μgmL^{-1} aqueous carbenicillin (Apollo Scientific, UK), inoculated with the transformed *E. coli* and left to stir overnight at 200 RPM and at a temperature of 37°C. The starter culture was added, along with 2 mL of 50 µg mL^{-1} aqueous carbenicillin, to 2 L of autoclaved LB media. This was left stirring at 200 RPM and 37°C until the optical density at λ = 600 nm was between 0.6 - 0.8. 20 mL of 23.8 mg mL^{-1} filter-sterilised solution of isopropylthiogalactosidase (IPTG) (Apollo Scientific, Japan) was then added to induce expression of eGFP. The growth culture was incubated overnight at 18°C.

The cultures were then centrifuged at 8000 g for 20 minutes using a Sorvall RC6 Centrifuge (Thermo Scientific, UK). The resulting green sediment was re-suspended in 50 mL of lysis buffer, which contained 50 mM NaH_2PO_4, 300 mM NaCl and 10 mM imidazole adjusted to pH 8 with NaOH. The cells were lysed using a Polytron PT2500 homogeniser (Kinematica, Germany), which released the contents of the cells, including the eGFP, into solution. To prevent proteolysis of the target cell, 100 µL of 100 mM phenylmethanesulfonylfluoride (PMSF) were added prior to the cell lysis step. The lysed sample was then centrifuged at 20000 g for one hour to remove any cell debris. The supernatant was then run through a metal ion affinity chromatography column, with nickel nitrilotriacetic acid (Qiagen, UK) as the immobile phase. Initially, lysis buffer was passed through the column to bind imidazole to the immobile phase, preventing any non-specific binding. The eGFP was then loaded onto the column *via* specific chelation of the polyhistidine tag to the nickel ions. The column was washed with 100% lysis buffer, and then the eGFP was eluted using elution buffer, consisting of 50 mM NaH_2PO_4, 300 mM NaCl and 250 mM imidazole adjusted to pH 8 with NaOH. The eluate purity was monitored by measuring the absorbance at λ = 280 nm, then the purified sample was then dialysed into deionised water using 12-14 kDa MWt cut-off dialysis tubing (Medicell International, UK). The sample was lyophilised at -40°C using a Free Zone freeze-drier (LabConco, USA) and then kept stored at -20°C for long-term usage.

Synthesis of myoglobin analogues

Apo-myoglobin and fluorescent myoglobin analogues were prepared using the following methods. A 100 µM ice-cold solution of equine heart myoglobin was acidified to pH 2.0 by the addition of 37% (v/v) hydrochloric acid (Sigma Aldrich, UK). An equivalent volume of 2-butanone was added to the myoglobin solution, which was transferred immediately to a separating funnel, kept at 4°C. The mixture was allowed to phase separate, with the haem porphyrin extracted into the organic layer above an aqueous layer containing apo-myoglobin. The aqueous phase was retained and put through two further solvent extractions. Excess 2-butanone was removed by three four-hour dialysis steps into 20 mM 4-(2-hydroxyethyl)-1-piperazineethanesulfonic acid (HEPES) (Sigma Aldrich, UK) buffer at pH 8.0. The resulting apo-myoglobin could then be added to a 1 mg mL^{-1} solution of protoporphyrin IX zinc (II) (Sigma Aldrich, UK) in dimethyl sulfoxide (DMSO) (Sigma Aldrich, UK), at a slight stoichiometric excess (1 mol protein : 0.85 mol protoporphyrin). The resulting zinc-substituted myoglobin was kept in the dark, to avoid the production of superoxide radicals.

Synthesis of glycolic acid ethoxylate 4-nonylphenyl ether

The glycolic acid ethoxylate 4-nonylphenyl ether surfactant was synthesised by the oxidation of the hydroxyl groups of a glycolic acid ethoxylate 4-nonylphenyl ether to yield a carboxylic acid terminus. 2 g of IGEPAL® CO-890 (Sigma Aldrich, UK) dissolved in 50 mL of deionised water was mixed with 30 mg 2,2,6,6,-tetramethyl-1-piperidinyloxyl (TEMPO) (Sigma Aldrich, UK), 50 mg sodium bromide (Sigma Aldrich, UK) and 5 mL of a sodium hypochlorite solution containing 10-15% available chlorine (Sigma Aldrich, UK). The solution was adjusted to pH 11 and then stirred for 24 hours before the reaction was quenched with the addition of 10 mL of ethanol. The solution was then adjusted to pH 1, before a solvent extraction with three 80 mL aliquots of chloroform (Sigma Aldrich, UK). The combined organic fractions were washed with three 80 mL aliquots of deionised water at pH 1, then dried under reduced pressure at 40°C. The resulting white solid was dissolved in 40 mL of ethanol at 65°C, and then recrystallised overnight at -20°C. The precipitate was isolated using vacuum filtration, dissolved in 20 mL ethanol and dried under reduced pressure at 40°C. The resulting product was a waxy white solid, similar to the reactant surfactant, with a yield of approximately 40%.

Cartilage digestion

Engineered cartilage constructs were transferred to individual 2 mL tubes (Eppendorf, UK), with a known mass, measured to 1/100th milligram accuracy using an AX105 balance (Mettler Toledo, Switzerland). These were lyophilised overnight using a ModulyoD freeze-drier (Fisher Scientific, UK) and then weighed to find the dry mass of the tubes containing the dry cartilage, which allowed the dry mass of the cartilage ($M_{cartilage}$) to be evaluated. The cartilage was processed by the addition of L of digestion solution, containing 2 mg mL^{-1} TCPK-treated trypsin (Sigma Aldrich, UK), 1 mM EDTA, 1 mM iodoacetamide (Sigma Aldrich) and 0.02 mg mL^{-1} Pepstatin A (Sigma Aldrich, UK) and 50 mM Tris-HCl (Sigma Aldrich, UK) at pH 7.2. The cartilage was heated overnight at 37°C in a Vortemp 56 shaking incubator (Labnet, USA). A further 125-500 µL of freshly-made digestion solution was added to the constructs, which were heated for three hours at 67°C in a shaking incubator and vortex-mixed at ten minute intervals for the first hour of incubation. The cartilage was then boiled for 15 minutes at 100°C to terminate the digestion, and centrifuged for two minutes at 15000 g. The supernatant, which contained the digested cartilage components, was transferred to fresh Eppendorf tubes for biochemical analyses. The pellet, which contained undigested scaffold, was washed with 500 µL of deionised water, centrifuged for two minutes at 15000 g, and then lyophilised overnight. The mass of the lyophilised scaffolds was calculated ($M_{scaffold}$) and used to evaluate the dry mass of the cartilage matrix (M_{matrix}) (**Equation 1**).

$$M_{matrix} = M_{cartilage} - M_{scaffold}$$ **Equation 1**

Glycosaminoglycan assay

A range of cartilage digest concentrations was prepared using a double series dilution into deionised water. The samples were mixed using a vortex mixer and added to a type 24 well plate as 20 µL triplicate aliquots. A range of eleven aqueous chondroitin sulfate (Sigma Aldrich, UK) concentrations were prepared, up to of 50 µg mL^{-1}, and 20 µL of each standard was added in duplicate to adjacent wells of the multi-well plate. A staining solution was prepared by mixing 8 mg of 1,9-dimethylene blue (Sigma Aldrich, UK) with 1.52 mg of glycine (Fisher Scientific, UK) and 1.185 mg of sodium chloride. 250 µL of the staining solution was added to the sample and standard solutions, and the absorbance at λ = 530 nm was measured immediately using a plate reader. The mean absorbance of each chondroitin sulfate dilution was used to construct a standard curve, which was used to estimate the unknown glycosaminoglycan concentrations. The glycosaminoglycan content was expressed as a percentage of the dry mass of the cartilage matrix.

Comparison of differences was tested using Wilcoxon non-parametric paired analysis, with two-tailed p-values of less than 0.05 considered significant.

Type I collagen assay

The inner wells of a Costar Immulon-2 high-binding 96-well plate #3455 (Appleton Woods, UK) were coated with 50 μL of a 0.04 mg mL^{-1} solution of AH23 peptide (CSFLPQPPQ; Pepceuticals, UK), dissolved in a 100 mM carbonate buffer at pH 9.2 made from sodium hydrogen carbonate (Fisher Scientific, UK) and sodium carbonate (BDH Laboratories, UK). Bubbles were removed with a syringe needle, and the plates were wrapped in cling-film and stored for three days at 4°C. The plates were washed three times using a wash solution (0.1% (v/v) Tween-20 (Sigma Aldrich) in PBS), and then tap-dried thoroughly. 60 μL of blocking solution (10 mg mL^{-1} BSA in PBS) was added to each coated well, and the plate was left to incubate for 30 minutes at room temperature. The plates were washed with the PBS/Tween, tap-dried thoroughly, dried at 37°C for 20 minutes, and wrapped in cling-film before incubation at 4°C. The inner wells of Costar 96-well plates #3798 (Appleton Woods, UK) were also blocked through the addition of 110 μL of BSA/PBS. The plates were left to stand at room temperature for 30 minutes, then washed with PBS/Tween and tap-dried thoroughly.

A solution comprised of 8 mg mL^{-1} SDS in Tris buffer was used to dilute nine AH23 peptide standards (3.9, 7.8, 15.6, 31.3, 62.5, 125, 250 and 500 $ngmL^{-1}$), as well as the digested cartilage samples. 100 μL of dilution solution was added to the first column of wells (B2-G2) of the #3798 plate as a negative control, and 50 μL of dilution solution was added to the second column of wells (B3-G3) as a maximum-binding control. 50 μL of each vortex-mixed standard was added, in duplicate, to the third, fourth and fifth columns (B4-G6), and 50 μL of each vortex-mixed sample was added, in triplicate, to the remaining inner wells (B7-G11). A 0.1% (v/v) solution of rabbit primary antibody against AH23 peptide (AH23-1319) was made using Tris/HCl buffer, with the addition of 4% (v/v) Triton-X100 (BDH Laboratories, UK). 5 μL of this antibody solution was added to the wells B3-G11. The plate was sealed and incubated overnight at 37°C.

50 μL aliquots were transferred from each well of the #3798 plate to the #3455 plate, in under 45 seconds, and the #3455 plate was sealed and left to stand at room temperature for exactly 30 minutes. The plate was washed with three times with PBS/Tween, tap-dried thoroughly and then further dried at 37°C for 30 minutes. 50 μL of antibody solution, prepared by diluting 5 μL of alkaline phosphatase (ALP) - conjugated goat anti-rabbit secondary antibody (Cambridge Biosciences, UK) in 5 mL of 10 mg mL^{-1} BSA/PBS with 5 μL of Tween-20, was added to the wells B2-G11. The plate was sealed and incubated at 37°C for two hours, then washed three times with PBS/Tween wash solution, once with deionised water then tap-dried thoroughly. 50 μL of substrate solution was prepared by dissolving one disodium p-nitrophenyl phosphate tablet (Sigma Aldrich, UK) in 10 mL of diethanolamine buffer, comprised of 12.3 mg magnesium chloride (Sigma Aldrich, UK) and 24 mL diethanolamine (Sigma Aldrich, UK) dissolved in 1 L of water at pH 9.8. The substrate solution as added to the wells B2-G11. The plate was incubated at 37°C for at least 10 minutes, then the absorbance at 405 nm was measured using a plate reader. The mean absorbance of each AH23 peptide concentration was used to construct a standard curve, which was used to estimate the unknown type I collagen concentrations. The type I collagen content was expressed as a percentage of the total cartilage matrix dry mass. Comparison of differences was tested using Wilcoxon non-parametric paired analysis, with two-tailed p-values of less than 0.05 considered significant.

Type II collagen assay

Type II collagen (Bioiberica, Spain) was dissolved in 1 mL of 100 mM carbonate buffer at pH 9.2 made from sodium hydrogen carbonate and sodium carbonate. This solution was thermally denatured at 80°C for

20 minutes, with vortex-mixing at five minute intervals, and then diluted to 0.06 mg mL^{-1} using carbonate buffer. 40 µL was added to the inner wells of a Costar Immulon-2 high-binding 384-well plate #3700 (Appleton Woods, UK), the bubbles were removed with a syringe needle, and the plates were wrapped in cling-film and incubated for three days at 4°C. The plates were washed three times using PBS/Tween and then tap-dried thoroughly. 50 µL of 10 mg mL^{-1} BSA in PBS was added to each coated well, and the plate was left to incubate for 30 minutes at room temperature. The plates were washed with wash solution, tap-dried thoroughly, dried at 37°C for 20 minutes, wrapped in cling-film, and stored at 4°C. The inner wells of a Costar 384-well plate #3702 (Appleton Woods, UK) were also blocked through the addition of 40 µL of BSA/PBS. The plates were left to stand at room temperature for 30 minutes, then washed with PBS/Tween and tap-dried thoroughly.

A dilution solution was prepared, comprising of 0.05 M Tris-HCl buffer adjusted to pH 7.6. This was used to dilute the seven CB11B peptide (GKVGPSGA[Hyp]GEDGR[Hyp]GP[Hyp]GP; Pepceuticals, UK) standards (0.5, 1.0, 2.0, 3.0, 4.0, 5.0 and 6.0 µgmL^{-1}) as well as the digested cartilage samples. 10 µL of dilution solution was added to the wells B2-C4 of the #3702 plate as a maximum-binding control, and 20 µL of dilution solution was added to the wells L2-O4 as a negative control. 10 µL of each vortex-mixed standard was added, in duplicate, to the wells E2-K4, and 10 µL of each vortex-mixed sample was added, in triplicate, to the remaining inner wells. A 0.17% (v/v) solution of monoclonal mouse primary antibody against denatured type II collagen (COL2-3/4) was made using Tris/HCl. 10 µL of this antibody solution was added to all wells, except the negative controls. The plate was sealed and incubated overnight at 37°C.

10 µL aliquots were transferred from each well of the #3702 plate to the #3700 plate, in under 45 seconds, and the #3700 plate was sealed and left to stand at room temperature for exactly 30 minutes. The plate was washed with three times with PBS/Tween, tap-dried thoroughly and then further dried at 37°C for 30 minutes. 10 µL of antibody solution, prepared by diluting 3 µL of ALP-conjugated goat anti-mouse secondary antibody (Cambridge Biosciences, UK) in 3 mL of 10 mg mL^{-1} BSA/PBS with 3 µL of Tween-20, was added to all the wells. The plate was sealed and incubated at 37°C for two hours, then washed three times with PBS/Tween, once with deionised water then tap-dried thoroughly. 10 µL of substrate solution, prepared by dissolving one disodium p-nitrophenyl phosphate tablet in 10 mL of diethanolamine buffer, was added to all the wells. The plate was incubated at 37°C for at least 10 minutes, then the absorbance at 405 nm was measured using a plate reader. The mean absorbance of each CB11B peptide concentration was used to construct a standard curve, which was used to estimate the type II collagen concentrations. The type II collagen content was expressed as a percentage of the total cartilage matrix dry mass. Where comparing samples, comparison of differences was tested using Wilcoxon non-parametric paired analysis, with two-tailed p-value of less than 0.05 considered significant.

Safranin O stain

The slides, containing the affixed sections, were immersed in xylene for two minutes to remove the paraffin wax, then rehydrated by two minute immersions in 100% ethanol, 90% (v/v) ethanol, 70% (v/v) ethanol and deionised water. A hydrophobic enclosure was created around each section using a PAP pen. 1 mg mL^{-1} fast-green FCF (Sigma Aldrich, UK) was added to each section for four minutes, then excess stain was removed by immersing the slides in 1% (v/v) acetic acid (Sigma Aldrich, UK). 5 mg mL^{-1} safranin O (Sigma Aldrich, UK) was added to each section for six minutes, then excess stain was removed by immersing the sections first in 95% (v/v) ethanol, and then in 100% ethanol. The slides were left standing in 100% ethanol for one minute, and then the hydrophobic enclosure was removed by a two minute immersion in xylene. The slides were left to dry for five minutes, then mounted on glass cover slips using a solution of di-n-butyl phthalate in xylene (DPX) (VWR, UK).

Immunohistochemistry

The cartilage sections, and positive controls of human tendon and cartilage, were immunostained using the following protocol. The slides, containing the affixed sections, were immersed in xylene for two minutes to remove the paraffin wax, then rehydrated by two minute immersions in 100% ethanol, 90% (v/v) ethanol, 70% ethanol (v/v) and deionised water. A hydrophobic enclosure was created around each section using a PAP pen. Two solutions were prepared for enzymatic antigen retrieval; 10 mg mL^{-1} hyaluronidase (Sigma Aldrich, UK) in PBS and 2 mg mL^{-1} pronase (Roche, Switzerland) solution in PBS. 200 µL of hyaluronidase solution was added to each section, and the slides were incubated in a humidified chamber at 37°C for 30 minutes. The enzyme solution was removed by immersing the slide in PBS for five minutes. 200 µL of pronase solution was added to each section, and the slides were incubated in a humidified chamber at 37°C for 30 minutes, followed by removal through immersion in a solution of PBS.

200 µL of 3% (v/v) hydrogen peroxidase solution was added to each section and incubated in a humidified chamber at room temperature for five minutes. The enzyme solution was removed by a five minute immersion in a solution of PBS. A TBS/Tween solution was prepared by dissolving 13.15 g of sodium chloride, 9.08 g Tris, and 0.75 mL Tween-20 in 1.5 L of deionised water (pH 7.5-7.6). 200 µL of blocking solution (30 mg mL^{-1} bovine serum albumin (BSA) in TBS/Tween) was added to each section, and the slides were incubated in a humidified chamber at room temperature for one hour. The blocking solution was removed with three ten-minute immersions in PBS. Primary antibody solutions were prepared in an antibody diluent comprising of 10 mg mL^{-1} BSA in TBS/Tween, 1% (v/v) goat anti-type I collagen (Cambridge Biosciences, UK) and 5% (v/v) goat anti- type II collagen (Cambridge Biosciences, UK), as well as an and isotype control of 5% (v/v) goat IgG Isotype (Santa Cruz, USA), were prepared in an antibody diluent. 70 µL of antibody solution or Isotype control was added to each section and incubated overnight in a humidified chamber at 4°C.

The excess antibody solution was removed by a 15 minute immersion in a high salt wash solution (25 mg mL^{-1} sodium chloride in TBS/Tween). This was followed by two further 15 minute incubations in TBS/Tween solution. 70 µL of secondary antibody solution, comprised of 0.5% (v/v) Vectastain biotinylated anti-goat antibody (Vector Laboratories, UK) in antibody diluent, was added to each section, and the slides were and incubated in a humidified chamber at room temperature for one hour. The excess antibody solution was removed by a 15 minute immersion in high salt wash solution, followed by two further 15 minute incubations in TBS/Tween solution. 200 µL of Vectastain avidin and biotinylated horseradish peroxidase macromolecular complex (ABC reagent) (Vector Laboratories, UK) was added to each section and the slides were incubated at room temperature for 30 minutes. The reagent solution was removed with three ten-minute immersions in TBS/Tween. 200 µL of ImmPACT diaminobenzidine tetrahydrochloride (DAB) substrate (Vector Laboratories, UK) was added to the sections, and the slides were incubated in a humidified chamber at room temperature for exactly ten minutes. The excess substrate solution was removed by rinsing the slides quickly in deionised water, followed by a five minute immersion in deionised water. The sections were then dehydrated by consecutive two minute immersions in 70%, 90% and 100% ethanol solutions. The hydrophobic enclosure was removed by a two minute immersion in xylene. The slides were left to dry for five minutes before being mounted on glass cover slips using a solution of DPX.

Optical microscopy and image analysis of stained tissue sections

All sections were imaged using a DMI 3000 optical microscope (Leica, UK). Safranin O images were then put through pixel counting image analysis using Image J software (National Institute of Health, USA). Images of cartilage sections from each patient were collated and converted to a 16 bit image. A mean

threshold was applied and external debris cleared, leaving discrete black and white images of the tissue sections. Particle analysis, from 0-infinity size and 0-1 circularity, was used to measure the quantity of the stained tissue sections. This was compared to the output from particle analyses of filled in sections, to obtain percentage values for the tissue and necrotic core. Comparison of differences was tested using Wilcoxon non-parametric paired analysis, with two-tailed p-values of less than 0.05 considered significant.

RNA extraction and qPCR analysis

Cartilage was engineered using 6 mm x 5 mm scaffolds and hMSCs that had been primed with 18 μM [Mb_C][S] or unlabelled hMSCs (control). After one, three, seven and 14 days, two cartilage constructs were sacrificed from each group. These constructs were washed with PBS and the inner segment of cartilage was isolated using a 3 mm biopsy punch (Stiefel Laboratories, USA). The segments were then pooled into four groups (primed inner, primed outer, unlabelled inner, unlabelled outer) in 2 mL tubes (Eppendorf, UK) for an RNA extraction using a Total RNA Purification Kit (Norgen Biotek Corp, Canada). Buffer RL was added to the tissue and mixed for ten minutes, before the addition of 200 μL of 100% ethanol. Each tube was vortex-mixed for ten seconds and then centrifuged at 200 g for ten minutes to pellet the scaffold. The supernatant from each sample was passed through a 20-gauge needle 20 times to shear any genomic DNA, before being transferred to spin columns. The spin columns were centrifuged at 14000 g for two minutes and the flowthrough was discarded. Three wash steps were performed by adding 400 μL of Wash Solution A and centrifuging the columns at 14000 g for two minutes, discarding the flowthrough after each wash. The columns were dried by a further centrifugation step at 14000 g for two minutes, and then placed into a fresh 1.7 mL elution tube. 50 μL of Elution Solution A was added to the column and centrifuged at 200 g for two minutes and then 14000 g for one minute. The same protocol was used to extract RNA from two control samples of plated cells (primed and unlabelled), however, in this case, lysis buffer and ethanol were added directly to the culture flask and the first centrifugation step was eliminated as there was no scaffold to pellet.

The RNA concentrations were measured using a DU530 spectrophotometer (Beckmann Coulter, USA). The one, three and seven day samples were then used to compare the amount of RNA in the inner and outer segments. cDNA was synthesised using a PrimeScript RT reagent kit (Takara, Japan) and a MJ Mini thermal cycler (BioRad Laboratories, USA) with a 37°C and 85°C annealing and denaturation temperature, respectively. A maximum of 2000 ng of RNA was used to synthesise 40 μL of cDNA, while no-template controls were prepared without any RNA. Quantitative real-time PCR was performed on total RNA using a 7500 real-time Applied Biosystems Detection System and 96-well human hypoxia Taqman ® array plates (Life Technologies, USA, Catalogue Number 4418735). For all PCR reactions, the cDNA template used was diluted to a final sample concentration of 50 ng μL^{-1} and three biological replicates were tested for validity. The reactions were incubated in Taqman array 96-well with the following cycling: 50°C for 2 minutes, 95°C for 10 minutes, followed by 40 cycles of 95°C for 15 seconds and 60°C for 1 minute. Ct values for all genes were normalised to the reference gene GAPDH. The relative expression was calculated using the arithmetic formula $2{-}\Delta\Delta CT$ and calibrated using the control or unlabelled samples. Genes with Ct values of 40 were considered to be not expressed. The fold change (FC) normalised to GAPDH expression was calculated for each replicate (i) of each gene (x) at time point (t) using Equation 2, where t0 is the plated control.

$$logFC = -[(x(i,t) - GAPDH(i,t)) - GAPDH(i,tO)]$$ **Equation 2**

The difference between the mean logFC values of the labelled and unlabelled groups was found for each gene, and comparison of differences was tested using a student t-test, with two-tailed p-values of less than

0.05 considered significant. All fold change calculation and statistical analysis was performed using R v3.1.0.

Flow cytometry studies

75,000 cells from four patients were plated in 4 mL of expansion medium in T25 flasks and cultured at 37°C for 72 hours. The media was aspirated, the cells primed with eGFP, [eGFP_C] and [eGFP_C][S], and then harvested with 1 mL of trypsin / EDTA solution at 37°C for five minutes. 4 mL of expansion media was added to the harvested cells, which were centrifuged at 1500 RPM for five minutes. The sediment was re-suspended in expansion media, and the number of harvested cells was counted. The cells were then centrifuged and re-suspended in PBS to remove any remaining growth media. Suspensions containing approximately 300,000 cells per mL and 0.004 mg mL^{-1} of propidium iodide were transferred to individual flow cytometry tubes, and analysed using a FACS Canto flow cytometer (BD Biosciences, USA) with FACS DIVA software (BD Biosciences, USA). The cell suspension was passed through the interrogation point at a rate of 100-300 events per second with a total of 10000 whole cell events measured. The side scatter area (SSCA), forward scatter area (FSCA), propidium iodide (PerCP-Cy5-5A) and eGFP fluorescence (FITCA-GFP) were measured using voltages of 290 V, 190 V, 490 V and 250 V, respectively.

FACS data were analysed using FlowJo software (Tree Star, USA), with unlabelled control cells used to define the gated areas used for all samples. Whole cells were defined by an FSCA vs SSCA gate, with data outside this region excluded as cell debris. The whole cells were further gated by defining an upper limit on the PerCPCy5A vs FITCA-GFP dot plot, and data above this limit were excluded as dead cells. The live cells were gated on a SSCA vs. FITCA-GFP plot, with data inside the region corresponding to non-fluorescent cells, and data outside the region corresponding to eGFP-positive cells. The gate for the unlabelled control sample was set to 0.5% eGFP positive. In addition, the mean fluorescence intensity for each sample was measured from the FITCA-GFP channel.

Mechanistic studies

The mechanistic studies required unconventional culture conditions, with added inhibitors or different types of growth media, and these conditions are listed below. To determine the endocytic pathway of eGFP internalisation, the inhibitors nystatin (Sigma Aldrich, UK), chlorpromazine hydrochloride (Sigma Aldrich, UK) and cytochalasin D (Sigma Aldrich, UK) were dissolved in DMSO (Sigma Aldrich, UK) to a concentration of 10 mM then freshly added to growth media to yield a final concentration of 30 μM. This supplemented growth media was used immediately after eGFP-priming. 1 μM [eGFP_C][S] was used during incubation and flow cytometry was performed 24 hours after priming.

To determine the role of cell surface proteoglycans on membrane adhesion, growth media was freshly supplemented with the 80 μM of sodium chlorate (Sigma Aldrich, UK) and used to culture the cells for four days prior to eGFP-priming. To determine the effect of membrane fluidity on membrane affinity, hMSCs were primed at 4°C , rather than 37°C. For these two studies, 6 μM [eGFP_C][S] was used and flow cytometry performed immediately after priming. These studies were carried out using cells from four patients, with a comparison of differences tested using a two-tailed paired t-test with a p-value of less than 0.05 considered significant.

Quantifying membrane uptake

In order to establish the equilibrium binding time between the protein-surfactant complexes and the cell membrane, hMSCs from a single patient were primed using 0.1, 1 and 6 μM solutions of [eGFP_C][S] for

in an incubation lasting either 10, 20, 30, 90 or 240 minutes. The cells were analysed using flow cytometry and a plot of mean fluorescence intensity against time was used to determine when equilibrium binding conditions were met. Following this, a second set of experiments were designed whereby hMSCs from two patients were primed for 240 minutes using [eGFP_C][S] concentrations of 0.5, 0.75, 1, 2, 4, 6, 10, 14 and 18 µM. The supernatant was analysed using fluorescence spectroscopy at an excitation wavelength of 488 nm, with the emission intensity monitored at 508 nm ($E_{508\ \mathrm{sample}}$). A standard curve was constructed using the emission intensities from [eGFP_C][S] solutions at known concentrations ($E_{508\ \mathrm{control}}$), which was used to estimate the difference in emission intensity (ΔE_{508}) (**Equation 3**).

$$\Delta E_{508} = E_{508\ \mathrm{control}} - E_{508\ \mathrm{sample}} \qquad \textbf{Equation 3}$$

These values were used to evaluate the concentration of [eGFP_C][S] delivered to the cell population at each concentration [P_{cell}] (**Equation 4**).

$$[P_{cell}] = \frac{\Delta E_{508} - c}{m} \qquad \textbf{Equation 4}$$

The incubation volume of protein-surfactant complexes (V), number of cells per flask (Q) and the Avogadro constant (N_A) was used to estimate the number of complexes per cell (N_{cell}) (**Equation 5**). Finally, these values were compared to a sample primed using normal conditions; i.e. 18 µM and 30 minutes.

$$N_{cell} = \frac{[P_{cell}]\, V\, N_A}{Q} \qquad \textbf{Equation 5}$$

Cytotoxicity studies

The cytotoxicity of the cell priming process was investigated using a (3-(4,5-dimethylthiazol-2-yl)-5-(3-carboxymethoxyphenyl)-2-(4-sulfophenyl)-2H-tetrazolium) (MTS) assay. 50,000 cells were plated in duplicate in 1 mL of expansion medium in a 24 well plate and cultured at 37°C for 24 hours. Under identical conditions, standard curve samples were established, in duplicate, with 0, 10,000, 20,000 and 50,000 cells per well. The cells were primed and then 0.5 mL of phenol-free expansion medium was added to each well, along with 100 µL of CellTiter 96 solution (Promega, USA). The cells incubated at 37°C for two hours, and then 100 µL was transferred, in triplicate, to a 96 well plate and the absorbance at 488 nm was measured using a plate reader. The absorbance intensities of the tested samples were averaged and then cell counts were extrapolated from the standard curve. These studies were performed using cells from four patients, with comparison of differences tested using Wilcoxon non-parametric paired analysis, with two-tailed p-value of less than 0.05 considered significant.

Proliferation studies

Cell counting was used to assay the effect of cell-priming on the proliferative capacity of hMSCs. 200,000 cells were primed with protein-surfactant complexes and the cells were harvested and counted using a haemocytometer. 100,000 cells from each sample were seeded into individual T25 flasks and cultured in expansion media. After one week, the cells were harvested and counted, and the process repeated for a second passage. Cell counts were converted into population doublings by taking a logarithmic quotient of the number of cells harvested (n_H) and the number of cells seeded (n_S) (**Equation 6**). All proliferation rates were normalised with respect to a control sample of cells incubated with PBS. These studies were

performed using cells from five patients and comparison of differences was tested using Wilcoxon non-parametric paired analysis, with two-tailed p-value of less than 0.05 considered significant.

$$Population\ Doublings = \frac{log\left(\frac{n_H}{n_S}\right)}{log(2)} \qquad \textbf{Equation 6}$$

Osteogenic differentiation of hMSCs

The inner wells of a 24 well plate were seeded with 37000 cells in 0.5 mL of expansion media, and the remaining outer wells were filled with sterile antifungal water containing 100 units mL^{-1} penicillin, 100 µg mL^{-1} streptomycin and 2.5 µg mL^{-1} amphotericin B (Sigma Aldrich, UK). The plates were incubated at 37°C for 24 hours, primed with [eGFP_C][S], and then returned to expansion media to equilibrate for 24 hours. Osteogenesis was induced by replacing the growth media with 0.5 mL of osteogenic differentiation media, comprising of Minimum Essential Medium containing $NaHCO_3$ (Sigma Aldrich, UK) with 100 units mL^{-1} penicillin, 100 µg mL^{-1} streptomycin, 2 mM GlutaMAX supplement, 10% (v/v) FBS and 50 µL mL^{-1} of freshly-added osteogenic supplement (R&D Systems, UK). An undifferentiated control was set up using an identical protocol, but without any added osteogenic supplement.

The differentiated and undifferentiated cells were grown for three weeks, with the media changed twice a week. At the end of this period, the differentiation media was aspirated, each well was washed with 500 µL of PBS, and then the cells were fixed by incubation with 500 µL of 70% (v/v) ice-cold ethanol at 4°C for one hour. The fixative was aspirated, then 500 µL of filtered Alizarin Red dye was added; this dye was prepared by stirring a 13.75 mg mL^{-1} aqueous solution of Alizarin Red (Sigma Aldrich, UK) overnight before adjusting the pH to 4.1 using a weak potassium hydroxide solution (Fisher Scientific, UK). After 5 minutes of staining at room temperature, the dye was aspirated and the cells were washed five times with 500 µL of PBS. Images were taken using a DMIL optical microscope (Leica, UK) with 10 X magnification.

Adipogenic differentiation of hMSCs

The inner wells of a 24 well plate were seeded with 7400 cells in 0.5 mL of expansion media, and the remaining outer wells were filled with sterile antifungal water containing 100 units mL^{-1} penicillin, 100 µg mL^{-1} streptomycin and 2.5 µg mL^{-1} amphotericin B. The plates were incubated at 37°C for 24 hours, primed with [eGFP_C][S], and then returned to expansion media to equilibrate for 24 hours. Adipogenesis was induced by replacing the growth media with 0.5 mL of adipogenic differentiation media, comprising of Minimum Essential Medium containing $NaHCO_3$ with 100 units mL^{-1} penicillin, 100 µg mL^{-1} streptomycin, 2 mM GlutaMAX supplement, 10% (v/v) FBS, and 10 µL mL^{-1} of freshly-added adipogenic supplement (R&D Systems, UK). An undifferentiated control was set up using an identical protocol, but without any added adipogenic supplement.

The differentiated and undifferentiated cells were grown for three weeks, with a media change twice a week. At the end of this period, the differentiation media was aspirated, each well was washed with 500 µL of PBS, and then the cells were fixed by incubation with 500 µL of 40 mg mL^{-1} paraformaldehyde at room temperature for 30 minutes. The fixative solution was prepared by dissolving 2 g of paraformaldehyde (Sigma Aldrich, UK) in 50 mL of PBS, heating this solution to 60°C, followed by the drop wise addition of dilute sodium hydroxide solution to give a clear solution. The fixative was aspirated and the cells were washed with 500 µL of PBS followed by 500 µL of 60% (v/v) isopropanol (Sigma Aldrich, UK). The cells were then stained with 400 µL of filtered Oil Red dye; this dye was prepared by stirring a 5 mg mL^{-1}

solution of Oil Red (Sigma Aldrich, UK) in isopropanol overnight, then diluting the solution to a concentration of 3 mg mL^{-1} using deionised water. After 30 minutes staining at room temperature, the dye was aspirated and the cells were washed briefly with 60% (v/v) isopropanol. Images were taken using a DMIL optical microscope (Leica, UK) with 10 X magnification.

Live-cell confocal microscopy

All confocal microscopy was carried out at the Wolfson Bioimaging Facility (University of Bristol). An Ultraview Spinning Disk confocal microscope (Perkin Elmer, UK) attached to a DMI 6000 inverted microscope (Leica, UK) was used for live-cell imaging of cells primed with [eGFP_C][S] or [Zn-Mb_C][S]. 50,000 cells were plated in 2 mL of expansion media in 35 mm diameter, glass-bottomed petri dishes. After 72 hours of culture, the cells were incubated with [eGFP_C][S] or [Zn-Mb_C][S], then transferred to phenol-free, low glucose (1000 mg dm^{-3}) DMEM containing pyridoxine-HCl and $NaHCO_3$ with 100 units mL^{-1} penicillin, 100 μg mL^{-1} streptomycin, 2 mM GlutaMAX supplement and 10% (v/v) FBS, buffered with 20 mM HEPES. The excitation source used was either an argon laser (488 nm) or a blue diode (405 nm) for cells labelled with [eGFP_C][S] and [Zn-Mb_C][S], respectively. Different exposure times and gains used to optimise each image, depending on the fluorescence of the sample. Three dimensional stacked images were obtained using a z-separation length of 0.25 μm. Images were captured and processed using Volocity software (Perkin Elmer, USA). Cell-loaded scaffolds were imaged one day after seeding using a TCS-SP2-AOBS confocal laser scanning microscope (Leica, UK). The scaffolds were washed three times with PBS, stained using a Live/Dead Cell Double Staining Kit (Sigma Aldrich, UK) and then imaged in phenol-free DMEM with 20 mM HEPES. The excitation sources used were an Argon laser (488 nm) and a HeNe laser (543 nm), to detect live and dead cells, respectively. Multiple images were captured across the centre of the scaffold, and these were stitched together using a demo version of AutoStitch software (University of British Colombia, Canada).

Raman microscopy

All Raman microscopy was carried out at Renishaw Old Town Site in collaboration with Dr Katherine Lau. Ovine MSCs cultured on a calcium fluoride substrate were primed with an 18 μM solution of either [Mb_C][S] or [apo-Mb_C][S]. The cells were fixed by incubation with 500 μL of 40 mg mL^{-1} paraformaldehyde at room temperature for 30 minutes. Raman spectra were collected using StreamLine HR confocal Raman imaging with an inVia Raman microscope (Renishaw plc, UK) coupled to a DM2500 microscope (Leica, UK). A 100X objective was used to focus a 532 nm excitation laser of approximately 5 mW in a scanning mode operation with 0.5 μm step size and approximately 0.4 μm lateral spatial resolution. Raman spectra were acquired at each pixel with a wavenumber range of ύ = 600 and 1800 cm^{-1} with a one second scan time period. The data was analysed using WiRE 4 software (Renishaw plc, UK), with cosmic ray removal and noise filtering followed by multivariate curve resolution – alternate least square (MCR-ALS) analysis. This gave the major components within the data set, with the characteristics of a component explained by the component resolved spectrum. The MCR-ALS scores were used to generate two-dimensional maps to reveal the distribution of the different components within individual cells.

Calculating oxygen availability

The number of protein-surfactant complexes delivered per cell (see Equation 4) was used as an estimate of the number of molecules of myoglobin per cell ($N_{Mb\,(cell)} \sim 5.9 \times 10^9$). With 1.2×10^6 cells seeded on each

scaffold, the tissue construct would contain an additional 7.1 x 10^{15} additional dioxygen molecules (**Equation 7**).

$$N_{O2\,(tissue)} = 1.2\;x\;10^{6}\;N_{Mb\,(cell)} \qquad \textbf{Equation 7}$$

Using the oxygen consumption rate of chondrocytes seeded on polymeric scaffolds (Q = 0.8 x 10^{-19} mol $cell^{-1}\,s^{-1}$) and the Avogadro constant (N_A) the additional oxygenation provided by [Mb_C][S] per cell (T_{cell}) was calculated as 34 hours (**Equation 8**). Note that this value relates to the supplementary oxygen supplied by [Mb_C][S] and does not include oxygen availability from surrounding media.

$$T_{cell} = \frac{N_{Mb\,(cell)}}{Q\;N_A} \qquad \textbf{Equation 8}$$

Mass spectrometry

The molecular mass of the native and cationised proteins was measured using Matrix-Assisted Laser Desorption / Ionisation Mass Spectrometry (MALDI-MS) (Applied Biosystems, UK). A DHAP / DAHC matrix was prepared by mixing a 20.3 mg mL^{-1} solution of 2,4-dihydroxyacetophenone (Sigma Aldrich) in ethanol was with aqueous diammonium hydrogen citrate (Sigma Aldrich) in a 3:1volume ratio. This matrix was then mixed with a 1 mg mL^{-1} aqueous protein sample and a 2% (v/v) trifluoroacetic acid solution in a 1:1:1 volume ratio. 3 µL aliquots were spotted onto a plate and dried at 45°C for 20 minutes. Multiple spectra were additively acquired between 5 and 50 kDa using FLEX Control software (Bruker, USA) and peaks were identified using FLEX Analysis software (Bruker, USA).

UV / visible spectroscopy

Samples were diluted in 10 mM of the corresponding buffer, and the absorbance was measured over the wavelength range λ = 200-700 nm using a Lambda 25 UV/visible spectrometer (Perkin Elmer, USA). The Beer-Lambert law (**Equation 9**) was used to calculate the protein concentration using the path length (L), the absorbance intensity (A_{280}) at λ = 280 nm and the extinction coefficients (ε_{280}) of 31270 and 13980 $M^{-1}cm^{-1}$ for eGFP and myoglobin, respectively (Protein Calculator, Scripps Research Institute).

$$A_{280} = \varepsilon_{280}\;C\;L \qquad \textbf{Equation 9}$$

Bicinchoninic acid assay

A bicinchoninic acid (BCA) assay was used to measure the concentration of proteins complexed with S1198 and S1783. 20 µL aliquots of protein were added in triplicate to a transparent 24-well plate. The equivalent cationised protein was used to construct a standard curve, with triplicate 20 µL aliquots of 0, 0.2, 0.4, 0.6, 0.8, 1.0 mg mL^{-1}. 200 µL of standard working reagent, a 50:1 ratio of reagents A and B (Thermo Scientific, UK) was added to each well, and the plate was incubated for 30 minutes at 37°C. The plate was removed from the incubator and left to cool for five minutes, before measuring the absorbance of each well at λ = 570 nm using a Mithras LB940 plate reader (Berthold Technologies, USA). The mean absorbance of the standards was used to construct a standard curve, which was used to calculate the concentration of the protein-surfactant complexes.

Dynamic light scattering

Particle size and zeta potential analysis was performed using a ZetaSizer Nano ZS (Malvern Instruments, UK) and ZetaSizer software (Malvern Instruments, UK). 15 µM samples were prepared in 10 mM of the corresponding buffer, centrifuged at 5000 RPM for five minutes and filtered using a 0.22 µm syringe filter. Zeta potential measurements were made after a 120 second equilibration with 10-100 runs per sample. Henry's equation was then used to fit the electrophoretic mobility (U_e) data, generating zeta potential (ζ) values as a function of the viscosity (η = 0.8872 cP) and dielectric constant (ε = 1.33) of the dispersant (**Equation 10**). As the protein samples were run in buffer conditions, the Smoluchowski approximation (*f*(Ka) = 1.5) was used as a general measure of Debye length (K) and particle radius (a).

$$Ue = \frac{2\varepsilon\zeta\, f(Ka)}{3\eta}$$ **Equation 10**

Size measurements were made after a 10 second equilibration, with the backscatter measured at 173°. The correlation data was fitted using a program written *in-house* for Igor Pro (WaveMetrics, USA). A plot of correlation (C) against time (t) was fitted to an autocorrelate function (**Equation 11**), based on a three particle system (X, Y, Z; e.g. monomers, dimers or larger aggregates) with each term comprised of a pre-exponential factor (X_1, Y_1, Z_1) between 0 and 1, as well as two cumulant terms (X_2 and X_3, Y_2 and Y_3, Z_2 and Z_3).

$$C = W + X_1 \exp(-2\, X_2\, t\, -\, X_3\, t^2) + Y_1 \exp(-2\, Y_2\, t\, -\, Y_3\, t^2) + Z_1 \exp(-2\, Z_2\, t\, -\, Z_3\, t^2)$$ **Equation 11**

The cumulant terms are dependent upon the scattering angle (θ), the refractive index of the medium (RI = 1.33) and the wavelength of the incident light (λ = 633 nm), contained within the scattering vector (Q) (**Equations 12-15**).

$$X_2 = D_x\, {Q_x}^2$$ **Equation 12**

$$Y_2 = D_y\, {Q_y}^2$$ **Equation 13**

$$Z_2 = D_z\, {Q_z}^2$$ **Equation 14**

$$Q = \frac{4\, \pi\, RI\, sin\, (\theta/2)}{\lambda}$$ **Equation 15**

In addition, the cumulant terms are also dependent upon the translational diffusion constant (D), which allows the mean hydrodynamic diameter (D_H) to be determined given a known thermal energy (k_BT = 4.11 J at 298 K) and dynamic viscosity (0.8772 x 10^{-3} cP) using the Stokes-Einstein Law (**Equation 16**).

$$D = \frac{k_B T}{3\, \pi\, \eta\, D_H}$$ **Equation 16**

The hydrodynamic diameter is normally distributed, allowing a polydispersity index (PDI) to be defined using the two cumulant factors (**Equation 17-19**).

$$PDI_X = \frac{X_3}{{X_2}^2}$$ **Equation 17**

$$PDI_Y = \frac{Y_3}{{Y_2}^2}$$ **Equation 18**

$PDI_Z = \frac{Z_3}{{Z_2}^2}$ **Equation 19**

Circular dichroism spectroscopy

Near and far-UV circular dichroism spectroscopy was performed using Beamline B23 running Global Works software (Olis, USA) at Diamond Light Source. Samples were diluted in the corresponding buffer, then scanned between λ = 240-340 nm and λ = 175-260 nm in 1 nm increments with 4 second integration times. The temperature was kept at a constant 24°C , except during thermal unfolding experiments, in which case the temperature was increased to 80°C or higher using a Peltier Temperature controller (Quantum, USA). Prior to each scan, the temperature was allowed to equilibrate for two minutes. All circular dichroism data was reported in terms of mean residue molar ellipticity (θ_{MR}), which was obtained by normalising the circular dichroism (ΔA) with respect to molar concentration (C), path length (L) and the number of residues (N) (**Equation 20**). DichroWeb software (Dichroweb, UK) was used to deconvolute far-UV ellipticity data into secondary structure fractions using the CDSSTR method and the reference database SP175 [1].

$[\theta]_{MR} = \frac{3298.2\ (\Delta A\ /C\ l\ N)}{10}$ **Equation 20**

Fluorescence spectroscopy

Samples were diluted to 0.01 µM using 10 mM of the relevant buffer, and fluorescence spectra were collected using a Fluoromax-2 fluorescence spectrometer (ISA Instruments, UK). eGFP samples were excited at λ = 488 nm, and emission collected over a wavelength range of λ = 450-700 nm. The excitation slit width was set at 1 nm, and the emission slit width was set at 5 nm. All data was normalised to arbitrary units using the sample concentrations.

GFP-Trap binding study

A 20 µL aliquot of GFP-Trap Beads (ChromoTek, Germany) were re-suspended in 500 µL of ice cold 10 mM tris(hydroxymethyl)aminomethane (Tris) (Sigma Aldrich, UK) buffer, which contained 0.5 mM ethylenediaminetetraacetic acid (EDTA (Sigma Aldrich, UK) and 150 mM sodium chloride. The suspension was centrifuged at 2700 g for two minutes at 4°C , and then the supernatant was discarded. This washing step was repeated a further two times, and then 30 µg eGFP in 500 µL of water was added to the sediment. The solution was then mixed for three hours at room temperature and then washed three times. An aliquot containing the beads were dispersed between a glass slide and cover slip and imaged using a SP5 confocal microscope (Leica, UK) attached to a DMI 6000 inverted microscope (Leica, UK), with a 0.1 second exposure time and 200 X saturation. Blocked agarose beads (Chromotek, Germany), lacking the GFP antibody, were used as a control experiment, with the same experimental protocols.

Equilibrium oxygen binding

4.5 µM solutions of native myoglobin and [Mb_C][S] in 20 mM HEPES at pH 8.0 were loaded into an anaerobic glove box (Belle Technologies, UK) and left stirring overnight to equilibrate to the hypoxic environment (2-3 ppm oxygen). The haem was reduced to a ferrous state by titrating sodium dithionite (Sigma Aldrich, UK) while monitoring the Soret absorption band using a USB2000+ spectrometer (Ocean Optics, USA). Aliquots of 20 mM HEPES buffer, kept at atmospheric oxygen tension, were added to the

reduced protein sample and the absorption intensity at the Soret band (433 nm for Mb, 426 nm for [Mb_C][S]) was measured. The average change in absorption intensity from three repeats was used to plot oxygen-binding curves, using a value of 0.258 mM as the concentration of molecular oxygen in water at 25°C and one atmosphere of pressure [2]. The data sets were then fitted to a model describing fraction of oxyferrous myoglobin (Φ) as a function of oxygen concentration $[O_2]$, accounting for the changes in free myoglobin [Mb] during oxygen binding (**Equation 21**). This allowed the oxygen dissociation constant (K_D) to be evaluated.

$$\Phi = \frac{[O_2]+[Mb]+K_D- \sqrt{([O_2]+[Mb]+[K_D])^2-4[O_2][Mb]}}{2}$$ **Equation 21**

Supplementary References

1. Lees, J. G., Miles, A. J., Wien, F. & Wallace, B. a. A reference database for circular dichroism spectroscopy covering fold and secondary structure space. *Bioinformatics* **22,** 1955–1962 (2006).

2. Carpenter, J. H. New measurements of oxygen solubility in pure and natural water. *Limnol. Ocean.* **11,** 264–277 (2013).
